# Crosstalk between genomic variants and DNA methylation in *FLT3* mutant acute myeloid leukemia

**DOI:** 10.1093/bfgp/elae028

**Published:** 2024-06-30

**Authors:** Bac Dao, Van Ngu Trinh, Huy V Nguyen, Hoa L Nguyen, Thuc Duy Le, Phuc Loi Luu

**Affiliations:** Hanoi Medical University, Hanoi, Vietnam; School of Biomedical Sciences, The University of Western Australia, Perth, Australia; The University of Texas MD Anderson Cancer Center, Houston, TX, United States; Health Innovation and Transformation Centre, Federation University, Victoria, Australia; Department of Population and Quantitative Health Sciences, UMass Chan Medical School; University of South Australia, Adelaide, Australia; Data Science Division, Tam Anh Research Institute (TamRI), 2B Pho Quang Street, Ward 2, Tan Binh District, Ho Chi Minh City 700000, Vietnam; Mathematics Department, Faculty of Fundamental Sciences, University of Medicine and Pharmacy at Ho Chi Minh City (UMP), 217 Hong Bang street, Ward 11, District 5, Ho Chi Minh City 700000, Vietnam

**Keywords:** *FLT3* mutant AML, DNA methylation, gene expression, WT1, TET2, Homebox gene family

## Abstract

Acute myeloid leukemia (AML) is a type of blood cancer with diverse genetic variations and DNA methylation alterations. By studying the interaction of gene mutations, expression, and DNA methylation, we aimed to gain valuable insights into the processes that lead to block differentiation in AML. We analyzed TCGA-LAML data (173 samples) with RNA sequencing and DNA methylation arrays, comparing *FLT3* mutant (48) and wild-type (125) cases. We conducted differential gene expression analysis using cBioPortal, identified DNA methylation differences with ChAMP tool, and correlated them with gene expression changes. Gene set enrichment analysis (g:Profiler) revealed significant biological processes and pathways. ShinyGo and GeneCards were used to find potential transcription factors and their binding sites among significant genes. We found significant differentially expressed genes (DEGs) negatively correlated with their most significant methylation probes (Pearson correlation coefficient of −0.49, *P*-value <0.001) between *FLT3* mutant and wild-type groups. Moreover, our exploration of 450 k CpG sites uncovered a global hypo-methylated status in 168 DEGs. Notably, these methylation changes were enriched in the promoter regions of Homebox superfamily gene, which are crucial in transcriptional-regulating pathways in blood cancer. Furthermore, in *FLT3* mutant AML patient samples, we observed overexpress of *WT1*, a transcription factor known to bind homeobox gene family. This finding suggests a potential mechanism by which WT1 recruits TET2 to demethylate specific genomic regions. Integrating gene expression and DNA methylation analyses shed light on the impact of *FLT3* mutations on cancer cell development and differentiation, supporting a two-hit model in AML. This research advances understanding of AML and fosters targeted therapeutic strategy development.

## Graphical Abstract

**Graphical Abstract:**
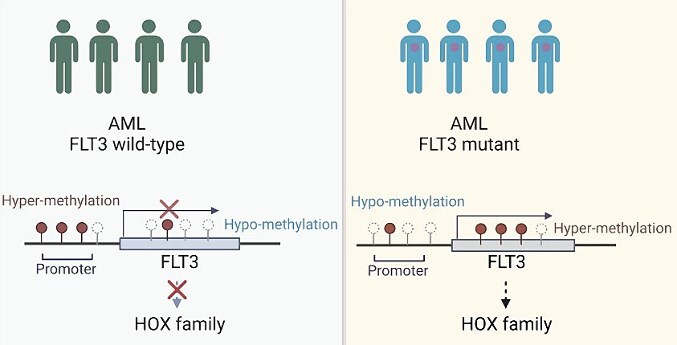

## Introduction

Acute myeloid leukemia (AML) stands as one of the most prevalent blood malignancies globally, accounting for 33% of blood cancers overall, and is characterized by the lowest survival rates (with a 5-year survival rate of 24%) [1, 2]. The advancements in next-generation sequencing technologies have unraveled the molecular aberrations in AML, categorizing them into several functional classes, such as signaling and kinase pathways, epigenetic modifiers, transcription factors, and tumor suppression [3] Among these, Class I (signaling and kinase pathways) exhibits the highest frequency of mutations, comprising nearly 50% of patients, with the *FLT3* mutation being the most prevalent (affecting one-third of patients) [4, 5]. *FLT3* encodes for a transmembrane receptor belonging to the tyrosine kinase receptor family, playing a pivotal role in the activation of hematopoietic stem cell proliferation, especially in cancer cells [6]. Numerous clinical trials have aimed to use drugs acting as FLT3 inhibitors to interact with these receptors and deactivate downstream signal transduction pathways [7–9]. Despite these efforts, remissions fail in 30%–40% of patients, leading to variable relapse rates of 30%–50%, attributed to diverse mechanisms of resistance, necessitating alternative therapeutic approaches [10].

DNA methylation, involving the addition of a methyl group to a DNA molecule, does not alter the nucleotide sequence of genes. However, changes in methylation status influence the start and end positions of each gene, playing a critical role in cancer pathogenesis [11]. Unlike genomic mutations, DNA methylation is a reversible process, making it a viable candidate for alternative therapies in cases of FLT3 inhibitor resistance. Furthermore, the pathogenesis of AML necessitates abnormalities in both cellular proliferation and cellular differentiation. *FLT3 *mutations are predominantly involved in the proliferation and survival of hematopoietic cells rather than in the latter ones [12]. This study was conducted to investigate the relationship between *FLT3* mutations and gene expression as well as DNA methylation, aiming to identify potential new therapies for FLT3 inhibitor resistance and shed light on the impact of *FLT3 *mutations on the differentiation of cancer cells.

## Results

### The association between genes expression and methylation on CpG sites of *FLT3* mutant AML patients

**Figure 1:**
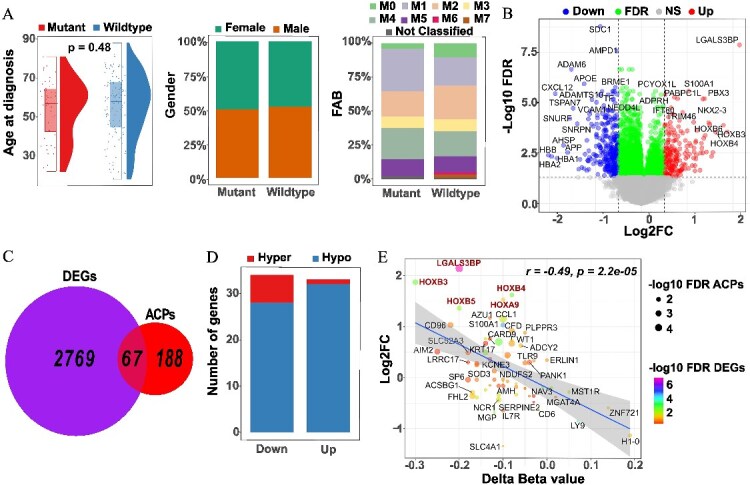
**Comprehensive analysis of *FLT3* mutant and wildtype groups, including patient characteristics, differentially expressed genes, and correlation patterns between gene expression and DNA methylation.** (**A**) Comparison of age, gender, and FAB subtypes between FLT3 mutant and FLT3 wildtype groups. (**B**) Differentially expressed genes between the two groups, with a false discovery rate (FDR) p-value adjustment (p < 0.05) and a Log2 fold change (Log2FC) threshold set at −0.5 and 0.5. (**C**) Overlapping of differentially expressed genes (DEGs) and their most differentially anti-correlated probes (ACPs). (**D**) Significant DEGs with corresponding significant ACPs. 67 DEGs were classified into down-regulated and up-regulated expression, while the ACPs in these 67 genes were categorized as either hyper-methylation or hypo-methylation. (**E**) Pearson correlation analysis between Log2 Fold Change in gene expression and Delta Beta methylated values. *(Down: Down-regulated, Up: Up: Up-regulated, NS: Not significant, FDR: False discovery rate, DEGs: Differentially expressed genes, ACPs: Anti-correlated probes, Hyper: Hyper-methylation, Hypo: Hypo-methylation).*

In the *FLT3 *mutant group comprising 48 samples, 43 mutations were identified as driver mutations, while the remaining 5 were classified as variants of uncertain significance. A majority of these mutations were in the form of inframe insertions, accounting for 27 out of the 48 mutations, and predominantly located in the juxta-membrane domain. The second most prevalent mutation type involved missense mutations (D835Y/E/H) within the Tyrosine kinase domain (Supplementary Fig. S1 and Supplementary File 1). No significant differences were observed in variables such as age at diagnosis, gender, FAB subtypes (The French-American-British classification of AML), or other clinical data between *FLT3* mutant and wild-type groups (Fig. 1A and Supplementary File 2).

Retrieving differentially expressed genes using cBioPortal, 2836 significant genes with a false discovery rate (FDR) < 0.05 were identified. These genes comprised 1020 up-regulated genes and 1816 down-regulated genes. However, when applying a threshold of |log2FC| > or = 0.5, 193 up-regulated genes and 318 down-regulated genes were retained (Fig. 1B).

In terms of CpG sites, significant differentially methylated probes associated with 255 unique genes were obtained from cBioPortal. For each gene, the single most anti-correlated probe (ACP) (Method section) was then selected for further analysis. The intersection of the 2836 differentially expressed genes (DEGs) and the 255 ACPs yielded 67 significant genes (Fig. 1C). Among these 67 genes, an almost equal number were divided into down-regulated and up-regulated genes, with 34 and 33, respectively. Notably, most of these genes exhibited hypo-methylated probes (60) as opposed to only 7 hyper-methylated probes (Fig. 1D).

A Pearson correlation analysis was conducted between Log2FC and Delta Beta values for these 67 genes. The Pearson correlation coefficient (r value) was −0.49 with a *P*-value of 2.2e-05, indicating a significant anti-correlated relationship between Log2FC and Delta Beta values. Of particular interest, several genes stood out, namely *LGALS3BP, HOXB3, HOXB4, HOXB5,* and *HOXA9*—with high expression and low methylation in the *FLT3* mutant group (Fig. 1E).

### Association of *FLT3* mutant groups with global hypo-methylation

**Figure 2:**
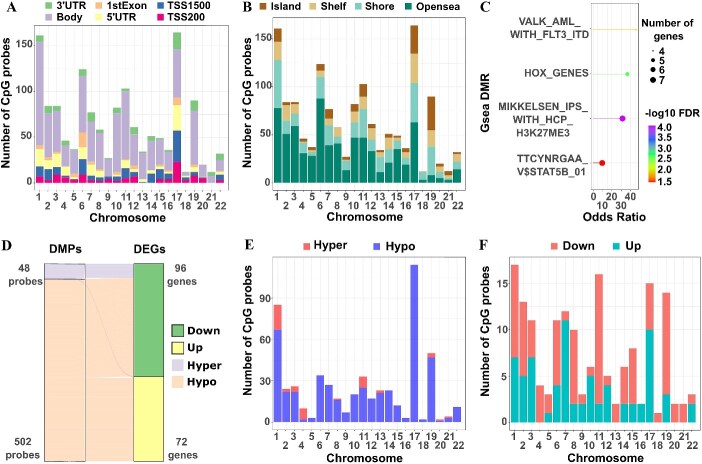
**Differentially methylated probes and region calling results.** (**A**) and (**B**) Distribution of differentially methylated probes (DMPs) in relation to their gene regions and CpG positions across chromosomes. (**C**) Gene set enrichment analysis of differentially methylated regions (DMRs) in mutant and wildtype groups. (**D**) The intersection of 168 DEGs with |log2FC| ≥ 0.5 and their corresponding DMPs (550 probes). (**E**) and (**F**) Analysis of 168 DMPs categorized by hyper-methylation or hypo-methylation and their association with down-regulated or up-regulated genes. *(TSS: Transcription start site, UTR: Untranslated region, DMRs: Differentially methylated regions, DMPs: Differentially methylated probes).*

It was observed that the most anti-correlated probes from cBioPortal did not capture a significant number of CpG sites within genes. Subsequently, a comprehensive analysis of differentially methylated probes and regions was conducted using the ChAMP tool in R [13]. The analysis unveiled 9060 significant differentially methylated probes (DMPs) and 48 significant differentially methylated regions (DMRs) (Supplementary Files 3 and 4). The negative correlation persisted upon comparing the log2FC and delta Beta values of 9060 DMPs (r = −0.23, *P* < 2.2e – 16) (Supplementary Fig. S2). Upon intersecting these 9060 significant DMPs with 2836 DEGs, a total of 1450 DMPs were identified within DEGs. Remarkably, the highest number of DMPs was found on chromosome 17, despite this chromosome having a relatively small representation on the 450 K methyl array (Fig. 2A and B and Supplementary Fig. S3). These DMPs on chromosome 17 were predominantly situated in the 5’UTR, 0–200 bases, and 200–1500 bases upstream of the transcription start site (TSS), while DMPs on other chromosomes were primarily located within the gene body, Chi-square test *P*-value <0.001 (Fig. 2A and Supplementary Fig. S4A and C). Additionally, a significant proportion of DMPs were situated in CpG islands and shelves compared with other chromosomes in open sea, Chi-square test *P*-value <2.2e-16 (Fig. 2B and Supplementary Fig. S4B and D). The integration of gene region and CpG positions was visualized (Supplementary Fig. S5).

Moreover, a Gene Set Enrichment Analysis pathway analysis was performed using data from MSigDB for DMRs [14]. This analysis revealed significant enrichment in the 'VALK_AML_WITH_FLT3_ITD' pathway, including genes: *HOXB5, LGALS3BP, HOXB2, HOXB3*, and 'HOX_GENES' pathway containing genes: *HOXB5, HOXA2, HOXA11, HOXB3, and HOXB2* (Fig. 2C and Supplementary File 5).

In a subsequent analysis, the integration of DMPs and DEGs was undertaken. However, this time, genes with |log2FC| greater than or equal to 0.5 was exclusively retained, resulting in 550 DMPs associated with 168 unique genes (Fig. 2D). Visualizing these 550 DMPs across chromosomes revealed that most of them were hypo-methylated (502), with the highest number of DMPs enriched in chromosome 17 (Fig. 2E). Regarding DEGs, among the 168 genes with DMPs, 72 were up-regulated, and 96 were down-regulated genes. Notably, a significant proportion of up-regulated genes were located in chromosomes 17 and 7 (Fig. 2F).

### Enrichment of significantly hypo-methylated CpG sites in the promoter regions of HOX genes

**Figure 3:**
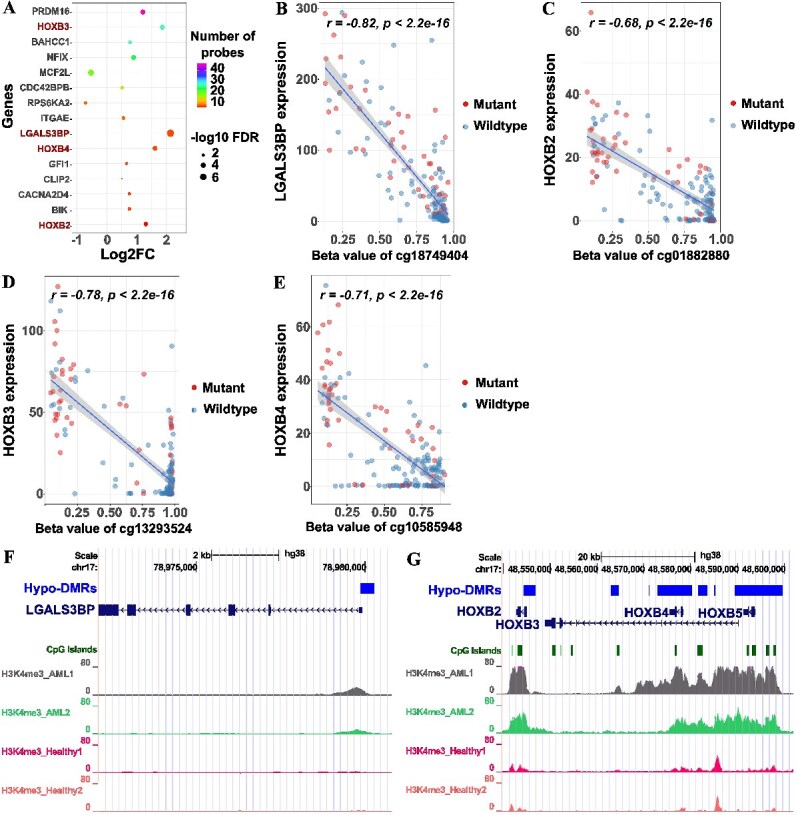
**Location of differentially methylated probes (DMPs) in chromosome 17 and their relationship with gene expression (A)** The top 15 genes with the highest number of DMPs are listed below along with their corresponding log2FC values. The significance of the log2FC gene expression is represented by the -log10 FDR (**B–E**) Pearson correlation analysis depicting the relationship between gene expression and Beta methylation value of *LGALS3BP* and HOXB gene family. (**F–G**) Genomic locations of DMRs in relation to H3K4me3 signals within *LGALS3BP* and HOXB gene family.

Upon visualizing the top 15 genes featuring the highest number of DMPs, the majority of them were up-regulated genes (13 genes), with only 2 being down-regulated genes. In the ranked table, *LGALS3BP, HOXB2, HOXB3, and HOXB4* were prominently featured and also had the highest log2FC values (Fig. 3A). Again, Pearson correlation analysis was conducted to explore the relationship between gene expression and Beta methylation values for these 4 genes. The results indicated highly significant Pearson correlation coefficients of −0.82, −0.68, −0.78, and − 0.71, respectively, with *P*-values < 2.2e-16 (Fig. 3B-E). These findings revealed a distinct separation between the two sample groups, with the mutant group exhibiting high gene expression coupled with low CpG methylation, while the wildtype group displayed the opposite trend. Additionally, other DMPs that were anti-correlated with gene expression for these four genes were also considered (Supplementary Fig. S6-S9).

Subsequently, we aimed to explore the precise locations of these significant DMPs within these gene regions. H3K4me3 signals from DCC Blueprint for two acute myeloid leukemia patients and two healthy individuals were incorporated. However, due to the reference genome differences (hg38 for H3K4me3 and hg19 for the 450 k array methylation), we employed the LiftOver tool from the UCSC Genome Browser to convert hg19 to hg38 and visualize the data [15]. *LGALS3BP* and the *HOXB* cluster were located on chromosome 17. DMRs hypomethylated in FLT3 mutant samples (Hypo-DMRs) were identified in the 5’UTR of these genes, overlapping with H3K4me3 signals. Furthermore, in the HOXB cluster, Hypo-DMRs also overlapped with CpG islands. The H3K4me3 signals were normalized in four samples, and signals from AML patients were notably higher than in healthy individuals (Fig. 3F-G). The integration of Pearson correlation values of DMPs and their genomic positions was illustrated in Supplementary Fig. S10. In addition to the *HOXB* cluster, the *HOXA* cluster exhibited up-regulation, as seen in the previous section. Therefore, we performed Pearson correlation analysis and visualized DMRs in this region. The *HOXA* cluster, located on chromosome 7, displayed a similar pattern to the *HOXB* cluster (Supplementary Fig. S11-S12).

### *WT1* recruits *IDH1/IDH2* and *TET2* for demethylation of *HOX* genes and their involvement in transcriptional regulatory pathways in cancer

**Figure 4:**
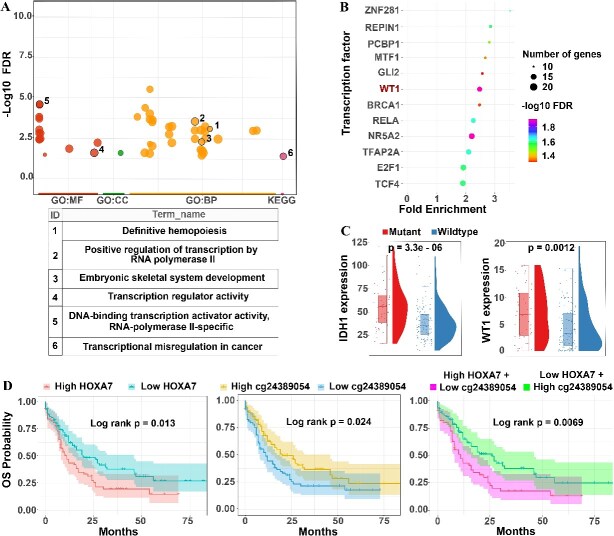
**Annotation of up-regulated genes, transcription factor finding and survival analysis (A)** Gene ontology analysis of the 72 up-regulated genes between the two groups. (**B**) Enrichment analysis was conducted on the promoter regions of 72 up-regulated genes to identify potential transcription factors. (**C**) A comparative analysis of *TET2* and *WT1* gene expression between the mutant and wildtype groups. (**D**) Kaplan–Meier survival analysis of two groups: up-regulated expression versus down-regulated expression and hyper-methylated versus hypo-methylated groups, showing their impact on overall survival (OS). *(MF: Molecular Function, CC: Cellular Components, BP: Biological Pathways (BP), KEGG: KEGG pathways).*

We have conducted a gene ontology analysis for 72 up-regulated genes in *FLT3* mutant group using g:Profiler (Supplementary file 6). A comprehensive analysis revealed that the Homeobox superfamily gene, including the *HOXB* cluster and *HOXA* cluster, was involved in these pathways. Additionally, it was observed that *WT1*, which was also up-regulated in the *FLT3* mutant group, was present in most of these pathways. Molecular Function and Biological Pathways consistently revealed enrichment in definitive hemopoiesis, embryonic skeletal system development, positive regulation of transcription, negative regulation of myeloid cell differentiation and the KEGG pathway related to transcriptional misregulation in cancer (Fig. 4A and Supplementary Fig. S13). Given the involvement of these 72 genes in transcriptional regulation pathways, we decided to investigate whether any genes among these 72 played a role as a factor in regulating the expression of other genes. ShinyGO v.75, using data from TRANSFAC and JASPAR, and GeneCards, using data from GeneHancer, were utilized to identify potential transcription factors [16, 17]. The analysis revealed that WT1 functions as a transcription factor binding to the promoter regions of other overexpressed genes (Fig. 4B and Supplementary file 7).

Previous studies have demonstrated that TET2 is regulated by IDH1/IDH2, while WT1 binds directly to TET2, recruiting it to WT1-specified genomic locations for the demethylation of targeted genes [18]. We have compared the gene expression of these 4 genes between FLT3 mutant and wildtype group and found that *IDH1* and *WT1* were also overexpressed in mutant group with a *P*-value of 3.3e-06 and 0.0012, respectively, while there was no significant difference in the gene expression of the other two genes (Fig. 4C and Supplementary Fig. S14). This finding supported the idea that *IDH1/IDH2 – TET2 – WT1* in the FLT3 signaling leads to the activation of the relevant genes.

Furthermore, *FLT3* also consistently exhibited higher expression across samples in the *FLT3* mutant group compared to the wildtype group. A hypo-methylated status was observed in the promoter region of *FLT3* (Supplementary Fig. S15). *FLT3* was also found to be involved in transcriptional misregulation in cancer and its promoter region was bound by WT1 according to Genecards.

We performed a survival analysis based on gene expression and CpG methylation value. The median value was used as the threshold to divide samples into 4 groups: those with values higher than the median were defined as overexpression and hyper-methylation, while those with values lower than the median were defined as down-expression and hypo-methylation. Giving *HOXA7* as an example, using Kaplan Meier method, samples that exhibited overexpression had a poorer overall survival (OS) probability than those with down-expression, with a log-rank test p-value of 0.013. Conversely, a reverse trend was observed in the differentially methylated probes (DMPs) within the *HOXA7* promoter region. Hyper-methylation in cg24389054 correlated with a better OS probability, with log-rank test *P*-values of 0.0224. Furthermore, combining gene expression and CpG methylation values provided enhanced predictive capability. High expression of *HOXA7* coupled with low methylation in cg24389054 was associated with a poor prognosis, with a log-rank test *P*-value of 0.0069 (Fig. 4D). Another probe also shared the similar characteristics was cg25727671 (Supplementary Fig. S16). The same survival analysis was performed with other up-regulated genes and their DMPs. *LGALS3BP* exhibited the same trends as *HOXA7*, while *RPS6KA2*, a down-regulated gene from section 3, displayed the opposite trends. Low gene expression and low methylation within *RPS6KA2* were associated with worse OS. Further analysis revealed that these DMPs belonged to a hypo-methylated region within the gene body of *RPS6KA2* (Supplementary Figs S17-S18).

To validate these results, gene expression data from the BEAT AML cohorts [19], of which included 618 samples comprising 203 *FLT3* mutant samples and 415 *FLT3* wildtype samples, were employed. It was noted that the same pattern was observed, with overexpression of the HOX gene family, *LGALS3BP*, *FLT3*, *WT1*, and down expression of *RPS6KA2* or *MCF2L* (Supplementary Fig. S19).

## Discussion

DNA methylation regulates gene expression by inhibiting the binding of transcription factors to DNA. An increasing in the level of DNA methylation reduce transcription and *vice versa* [11]. Our study showed that in the *FLT3* mutant AML patients, there was a significant hypo-methylated status enriched in DEGs, specifically *HOX* genes family, consistent with increased transcriptional levels of these genes. Furthermore, the impact of DNA methylation on gene expression is dependent on the methylation positions, whereby reducing methylated status in the promoter region will enhance transcriptional activity while decreasing methylation in the gene body region (not promoter) brings the opposite effect [20]. The current study showed that the demethylated positions coincide with the peaks of H3K4me3 from ChIP-seq—which indicates the promoter region of the genes. On the other hand, the hyper-methylation sites are located in the gene body instead of the promoter region, which leads to activating these genes' transcriptional activity [21]. In addition, enrichment of demethylated positions was mostly found in TSS1500 or TSS200 regions, which once again indicates the over activity of promoter regions in these genes at AML.

Among the 72 overexpressed genes identified in our study, apart from the* HOX* gene families associated with ANTP-class homeodomains—specifically the *HOXA* gene family on chromosome 7 and the *HOXB* gene family on chromosome 17—we detected *NKX2–3* within the NKL subclass, along with *IRX3, MEIS1*, and *PBX3* within the TALE-class homeodomains (Supplementary file 6). All these homeobox genes were found to be linked to significant cancer-enriched pathways (Supplementary Fig. S13). This discovery is consistent with prior research that has established an association between hematopoietic leukemia and the dysregulation of homeobox genes. For instance, expression of all *HOXA* cluster members was predominantly observed within myelomonocytic cells [22]. Recent studies have shown the activation of *HOXA9* and *HOXA7 via* proviral integration in a mouse model of myeloid leukemia [23]. Moreover, increased expression of *HOX* genes in hematopoietic cells has been suggested to markedly disrupt the differentiation of various cell lineages, potentially contributing to the onset of leukemogenesis [24]. *IRX3* has been validated to be overexpressed alongside *HOXA* genes in AML and has also been linked to the blockade of myelomonocytic differentiation [25, 26]. Our current study further corroborates the elevated expression levels of the *HOX* gene family, particularly the *HOXB* gene cluster, *NKX2–3*, and *MEIS1* in *FLT3-ITD* mutation cases [27].

In our investigation, *LGALS3BP (known as* Galectin-3 binding protein), was found to be overexpressed in *FLT3* mutation cases and was associated with a poorer prognosis. Additionally, a decrease in methylation at CpG sites within the *LGALS3BP* promoter region was linked to a lower overall survival probability (Supplementary Fig. S17). Numerous studies have indicated that markedly increased levels of *LGALS3BP* in serum or tumor tissues have been linked to adverse clinical outcomes in various malignancies, such as neuroblastoma, glioblastoma, breast cancer, and endometrial cancer [28–32].

*FLT3* mutations in acute myeloid leukemia are divided into two major types, *FLT3-ITD* (internal tandem duplication in the juxta-membrane domain) and *FLT3-TKD* (point mutations or deletion tyrosine kinase domain) [5]. In our study data, 26/48 samples were *FLT3-ITD* while the remaining were *FLT3-TKD*. Both of them cause self-dimerization and phosphorylation of tyrosine kinase receptors, regardless of interaction with the ligand [33]. As a result, mutant tyrosine kinase receptors constitutively activate a variety of intracellular signaling pathways such as RAS/MAPK, and PI3K/AKT leading to uncontrolled cell proliferation and resistance to apoptosis [34]. Our study revealed a decrease in DNA methylation in the promoter region and an increase in DNA methylation in the gene body of *FLT3* within the *FLT3 *mutant group, which may contribute to its heightened expression.

*WT1* was first known as a tumor suppressor gene in Wilms tumor, regulating the differentiation of renal and gonadal cells [35]. Surprisingly, *WT1* has been revealed to be overexpressed in several tumor tissues such as ovarian and breast cancer [36]. In hematologic malignancies, WT1 report as an oncogene and is overexpressed in most AML specimens, especially with *FLT3* mutation samples and associated with poor prognosis [37–39]. The activation of the PI3K-AKT pathway has been demonstrated to upregulate *WT1* mRNA levels in leukemia cells [40]. WT1 functions as a transcription factor, binding to specific DNA regions and recruiting TET2 to modulate DNA methylation [18, 41]. Based on previous studies and our analysis, we propose that WT1 could potentially act as a DNA binding factor for the Homeobox superfamily genes and FLT3, thereby recruiting TET2 to these regions. In summary, *FLT3* mutations might induce the transcription of *WT1*  *via* the PI3K-AKT pathway. This cascade of events contributes to definitive hematopoiesis, embryonic process activation, transcriptional misregulation in cancer, and resistance to differentiation [42].

These findings provide insights into a two-hit model in AML, indicating the necessity for inhibiting differentiation besides cellular proliferation in *FLT3* mutations [43]. Recently, *WT1* with increased expression in cancer cells in general and particularly in leukemia, has been considered as an antigen to apply immunotherapy [44]. Our finding might also pave the way for alternative treatment options when it comes to FLT3 inhibitor resistance, WT1 might be considered an efficient target for further research.

In summary, our hypothesis posited a scenario of overall reduced DNA methylation coupled with the overexpression of misregulation in cancer pathway-related genes in *FLT3* mutant acute myeloid leukemia. Additionally, our findings suggest the potential clinical application of alternative genes and DNA methylation patterns as alternative therapeutic options and as predictors of prognosis.

## Limitations

The utilization of data-mining-driven approaches in tumor biology inherently imposes constraints, particularly concerning the hypotheses put forward in the absence of definitive validation studies. The reliance on experimental methodologies conducted by other laboratories inevitably constrains the breadth of conclusions that can be drawn from these analyses. Though efforts were made to validate gene expression using the BEAT AML cohort, the scarcity of analogous DNA methylation datasets limited the ability to cross-validate the findings.

The reliability of WT1 binding sites could be significantly bolstered with additional supporting evidence from ChIP-seq experiments, ideally conducted in leukemia cell lines or extracted from tumor cells of affected patients. Furthermore, for a more comprehensive understanding of the signaling pathway connecting *FLT3* mutation to *WT1* overexpression, future experiments involving the knock-out of *FLT3* and PI3K/AKT would be instrumental.

## Methods

Our project utilized various computational tools and software to perform genomic profiling, differentially expressed genes calling of *FLT3 mutant* and *FLT3 wildtype* acute myeloid leukemia samples, and data visualization. Table 1 provides a list of the relevant software and web databases and briefly explains their contribution to this publication.

**Table 1:** Software and databases

Software/Database	Description	Location
GDAC Firehose	mRNAseq samples Array methyl 450 K samples	http://firebrowse.org/ (Accessed on 10 March 2022)
cBioPortal [45]	Genomics profiling Differentially expressed genes calling Mutual exclusive test Clinical information	https://www.cbioportal.org/ (Accessed on 10 March 2022)
ShinyGO [16]	Transcription factors analysis	http://bioinformatics.sdstate.edu/go75/ (Version 0.75. Accessed on 10 September 2023)
GeneCards [17]	Transcription factors analysis	https://www.genecards.org/ (Accessed on 10 September 2023)
Ensembl [46]	H3K4me3 signals	https://www.ensembl.org/index.html (Accessed on 10 March 2022)
UCSC Genome Browser [15, 47]	Genomic visualization Convert genome coordinates between assemblies	https://genome.ucsc.edu/ (Accessed on 24 August 2022)
g:Profiler [48, 49]	Functional enrichment Gene ontology analysis	https://biit.cs.ut.ee/gprofiler/gost (Accessed on 10 September 2023)
ChAMP [13]	Differentially methylated region calling Gene Set Enrichment Analysis	https://bioconductor.org/packages/release/bioc/html/ChAMP.html (Version 2.32.0. Accessed on 10 March 2022)
Biorender	Biological figure generation	https://www.biorender.com/ (Accessed on 10 September 2023)
Inkscape	Scientific figures editing	https://inkscape.org/ (Accessed on 10 September 2023)

### Data source and collection

The primary dataset was obtained from the TCGA-LAML (Acute Myeloid Leukemia) repository, publicly available *via* GDAC Firehose. This dataset comprised RNA sequencing data from 179 samples and 450 K methyl array data from 194 samples. Additional* FLT3* mutant status information for 197 samples was sourced from cBioportal. Subsequently, following the merge, the data was categorized into a subset of 173 samples, of which 48 were identified as having *FLT3 *mutant status, while 125 samples displayed wild-type *FLT3* status.

Four H3K4me3 ChIP-seq tracks are available from Blueprint through Ensemble: Two of healthy individuals (BM030613 and BM060814) (Neutrophils) and two of acute myeloid leukemia (UMCG00014 and UMCG00018) (Bone marrow).

### Differential analysis

Differentially expressed genes (DEGs) and differentially methylated probes (DMPs) between *FLT3* mutant and wild-type groups were commenced within the cBioportal platform. The log2-fold change (Log2FC) transformation was employed to quantify disparities in gene expression, while delta-Beta transformation assessed CpG methylation variances between the distinct groups.

For each gene, one DNA methylation probe was selected based on the correlation with its mRNA expression level. If multiple probes for a gene were available, the probe that had the most negative correlation value, was selected, and noted as most anti-correlation probes (ACPs). Overexpressed genes were delineated by a Log2FC (Log2 fold change) of ≥0.5, whereas downregulated genes were characterized by a Log2FC of ≤ 0.5.

### Extensive methylation analysis

A comprehensive analysis of differentially methylated probes (DMPs) and regions was conducted using ChAMP tools. The limma package was employed for DMP analysis, while the Bumphunter method was utilized for differentially methylated regions (DMRs). DMRs were defined using the following criteria: A DMR was required to contain at least seven probes, and the maximum distance allowed between consecutive probes within a DMR was set to 300 base pairs (bp).

To reduce potential bias that may arise during analysis, DMPs that were situated in sex chromosomes (X and Y) were excluded from our analysis.

Probes with a delta-Beta value < −0.2 were classified as hypomethylated, whereas those with a delta-Beta value >0.2 were deemed hypermethylated.

DMRs exhibiting a delta Beta value < −0.5 were categorized as hypomethylated regions, whereas those with a delta Beta value >0.5 were classified as hypermethylated regions. DMPs were annotated in relation to genomic regions and CpG locations using data from Illumina 450 k Bead Arrays within the ChAMP tool.

For the Enrichment Analysis of DMRs, we integrated the missMethyl package into the ChAMP toolset to mitigate biases arising from the distribution of methylation probes. Data utilized for this analysis was sourced from the Molecular Signatures Database (MsigDB).

### Genomic visualization

For a visual representation, DMRs and DMPs coordinates were converted from the hg19 to hg38 reference genome using the LiftOver utility in the UCSC Genome Browser. These DMRs and DMPs were then superimposed with H3K4me3 data for comprehensive genomic visualization.

### Functional enrichment and transcription factor analysis

The functional implications of our findings were explored using gene ontology and functional enrichment analyses, utilizing g:Profiler. Additionally, potential transcription factors binding to promoter regions (−2000 bp to +200 bp relative to the TSS) were identified by retrieving information from the TRANSFAC and JASPAR databases *via* ShinyGO v0.75 and from GeneHancer *via* GeneCards.

### Clinical attributes

A comprehensive comparative analysis of clinical attributes between the groups was conducted. Survival analysis was performed by segregating groups based on either gene expression or methylation beta value, using the Kaplan–Meier method with a log-rank *P*-value threshold of <0.05.

### Statistical analysis

Standard analytical packages, statistical analyses, and predefined software parameters were employed in data collection from cBioPortal.

A Pearson correlation analysis was conducted to examine the association between gene expression and DNA methylation.

Data were compared between the two groups (*FLT3* mutant and *FLT3* wildtype groups) using Wilcoxon sum rank tests for continuous variables or Chi-square tests for categorical variables. Multiple comparisons were subjected to the Benjamini–Hochberg FDR correction with a stringent threshold of FDR < 0.05.

All analyses were performed using R (4.2) software.

**Key Points**

- Revealing complex interactions between gene mutations, expression, and DNA methylation in *FLT3 *mutant AML.
- Uncovering a global hypo-methylated status in key genes, particularly within the Homeobox gene family promoter region.
- Unveiling a potential mechanism involving *WT1* overexpression and recruitment of TET2 to demethylate specific genomic regions.
- Supporting two-hit model including cellular proliferation and differentiation in leukemia.

## Supplementary Material

Supplementary_Figure_elae028

Supplementary_file_1_elae028

Supplementary_file_2_elae028

Supplementary_file_3_elae028

Supplementary_file_4_elae028

Supplementary_file_5_elae028

Supplementary_file_6_elae028

Supplementary_file_7_elae028

Supplementary_material_elae028

## Code availability

Data and Jupyter notebooks containing R code for running and generating figures are available at https://github.com/bacdao/LAML. Genomic locations of DMRs and DMPs in relation to H3K4me3 signals are available at public session https://genome.ucsc.edu/s/bacdao/LAML.

## Author Biographies

**Bac Dao**, is a medical professional and researcher specializing in medical genetics. He earned a Master of Science in Medical Genetics from Hanoi Medical University and is currently a PhD candidate at The University of Western Australia. His primary research areas include cancer, epigenomics, and bioinformatics. He was granted a prestigious NIH grant for the project titled “Training Program for Strengthening Research Capacity in Non-Communicable Diseases in Vietnam (TSORC-NCDs-VN)” [D43TW012188]. His current research endeavours focus on utilizing machine learning models to advance precision medicine, with the goal of improving personalized treatment strategies for patients.

**Dr. Van Ngu Trinh** is a Postdoctoral research fellow at the Department of Experimental Radiation Oncology at The University of Texas MD Anderson Cancer Center. He obtained his PhD at the SoonChunHyang Institute of Medi-Bio Science, SoonChunHyang University, South Korea. Currently, Dr. Trinh’s research is centered on the tumor microenvironment, leveraging multi-omics approaches to advance the field of immunotherapy, with a particular emphasis on developing personalized cancer therapies. With a strong background in cancer biology and cancer metastasis, his work aims to unravel the complex interactions within the tumor microenvironment and integrate various omics data to enhance the efficacy and precision of immunotherapeutic strategies, ultimately contributing to improved outcomes for cancer patients.

**Dr. Huy Nguyen** is a clinical doctor and PhD specializing in biostatistics, epidemiology, and research methods. Since 2021, he has joined as a biostatistician at Federation University. Through his career, he has been a successful co-developer, coordinator, senior researcher and biostatistician of more than 30 various projects, including those funded by NIH. He is active in research with over 90 publications in high-impact journals, including Lancet. Some of his research findings informed health policies and practice, such as tobacco control law in healthcare settings and HIV/AIDS prevention and control strategies in Vietnam.

**Dr. Nguyen** is an Associate Professor in epidemiology who has more than 20 years of experience in public health, clinical research, and global health. She has received grant funding support from the NIH to conduct several observational studies and randomized controlled trials in Vietnam. She served as the Principal Investigator for a study aiming to establish a population-based registry for patients hospitalized with acute myocardial infarction in northern and central provinces of Vietnam. She was a key investigator on an NHLBI-funded study examining the effectiveness of the Storytelling intervention in improving hypertension control in rural settings of Vietnam. She is also MPI for an NCI-funded study evaluating the effectiveness of a motivational text message intervention in increasing the use of quit lines and smoking cessation in People Living with HIV in Vietnam. She is Multi-PI for a Fogarty-funded training program in non-communicable disease in Vietnam, in which a new Master in Epidemiology in English and a Postdoctoral fellowship program have been established at Hanoi Medical University. She is also the contact PI for a newly NLHBI-funded study evaluating the effectiveness of multi-component intervention in improving hypertension control in urban settings of Vietnam. She serves as the instructor for several courses offered at the University and a mentor to several Masters, Ph.D, and postdoctoral trainees. She has served on scientific review panels on global health at the NIH.

**Thuc D. Le** is an associate professor at UniSA STEM, University of South Australia. He was an ARC DECRA fellow (2020-2022) and an NHMRC ECR fellow (2017-2019). His research interests include causal AI methods and their applications in different areas.

**Dr. Loi** is currently head of the Data Science Division, Tam Anh Research Institute, Viet Nam. Before moving back to Vietnam in 2023, Dr. Loi was a medical researcher at Garvan Institute of Medical Research, Sydney Australia from 2015 to 2023. He has developed a long-term interest in clinical epigenetics and genomics using the next-generation sequencing (NGS) technologies since his Bioinformatics Master training at the Max Planck Institute for Informatics (2011) and doctoral at the Max Planck Institute for Molecular Biomedicine (2014), Germany. Based on the NGS, Loi has built multiple bioinformatics tools and wet-let protocols for a wide range of studies from mechanism studies to translational research. After moving to Garvan Institute of Medical Research (Sydney, Australia), Loi established his own research topic on translational studies including the roles of genomics, DNA methylation on gene expression regulation, pharmaco-epigenomics and epigenetics biomarker development for prognosis, diagnosis and disease progression monitoring in breast, prostate cancer, liver, lung cancer, and also rare genetic diseases.

## Notes

**Biographical note:** We are a dedicated research team exploring genetic and epigenetic dimensions of cancer, with a specific focus on acute myeloid leukemia (AML). Our passion drives innovative approaches to understanding and treating this disease.
