# Supplementary material for: Crosstalk between genomic variants and DNA methylation in FLT3 mutant acute myeloid leukemia
Source: Brief Funct Genomics. 2024 Jun 30;24:elae028. doi: 10.1093/bfgp/elae028 (PMC11735749; doi:10.1093/bfgp/elae028)
Supplement: Supplementary_Figure_elae028 [file supplementary_figure_elae028.docx]

**Supplementary Figure S1**


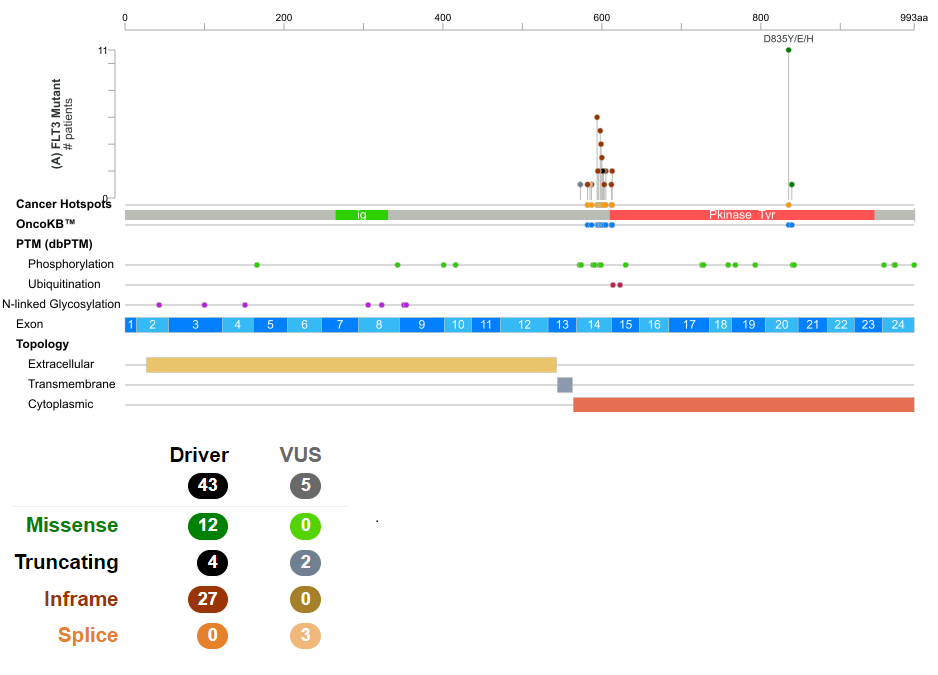


Lolipop plot illustrates the FLT3 mutation types in relation to genomic position, Cancer Hotspots, Post Translational Modification (PTM), and Topology (Adapted from cBioportal).

Mutation types and corresponding color codes are as follows:

- **Missense Mutations** (putative driver)
- **Missense Mutations** (unknown significance)
- **Truncating Mutations** (putative driver): Nonsense, Nonstop, Frameshift deletion, Frameshift insertion, Splice site
- **Truncating Mutations** (unknown significance): Nonsense, Nonstop, Frameshift deletion, Frameshift insertion, Splice site
- **Inframe Mutations** (putative driver): Inframe deletion, Inframe insertion
- **Inframe Mutations** (unknown significance): Inframe deletion, Inframe insertion
- **Splice Mutations** (putative driver)
- **Splice Mutations** (unknown significance)
- **Other Mutations** (putative driver): All other types of mutations
- **Other Mutations** (unknown significance): All other types of mutations

**Supplementary Figure S2**

**
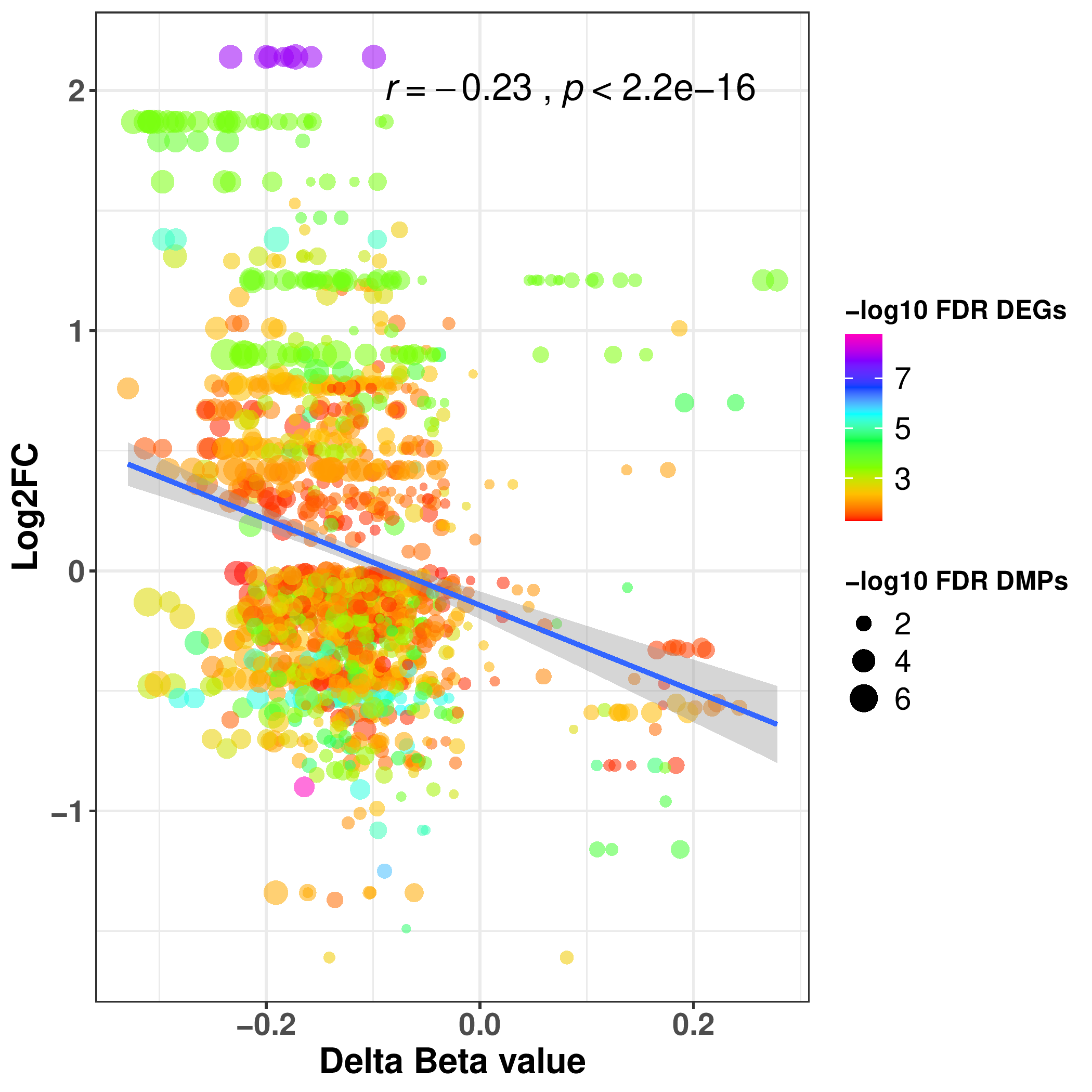
**

Pearson correlation analysis between Delta Beta methylated values of 9060 DMPs and their corresponding Log2 Fold Change in gene expression.

**Supplementary Figure S3**


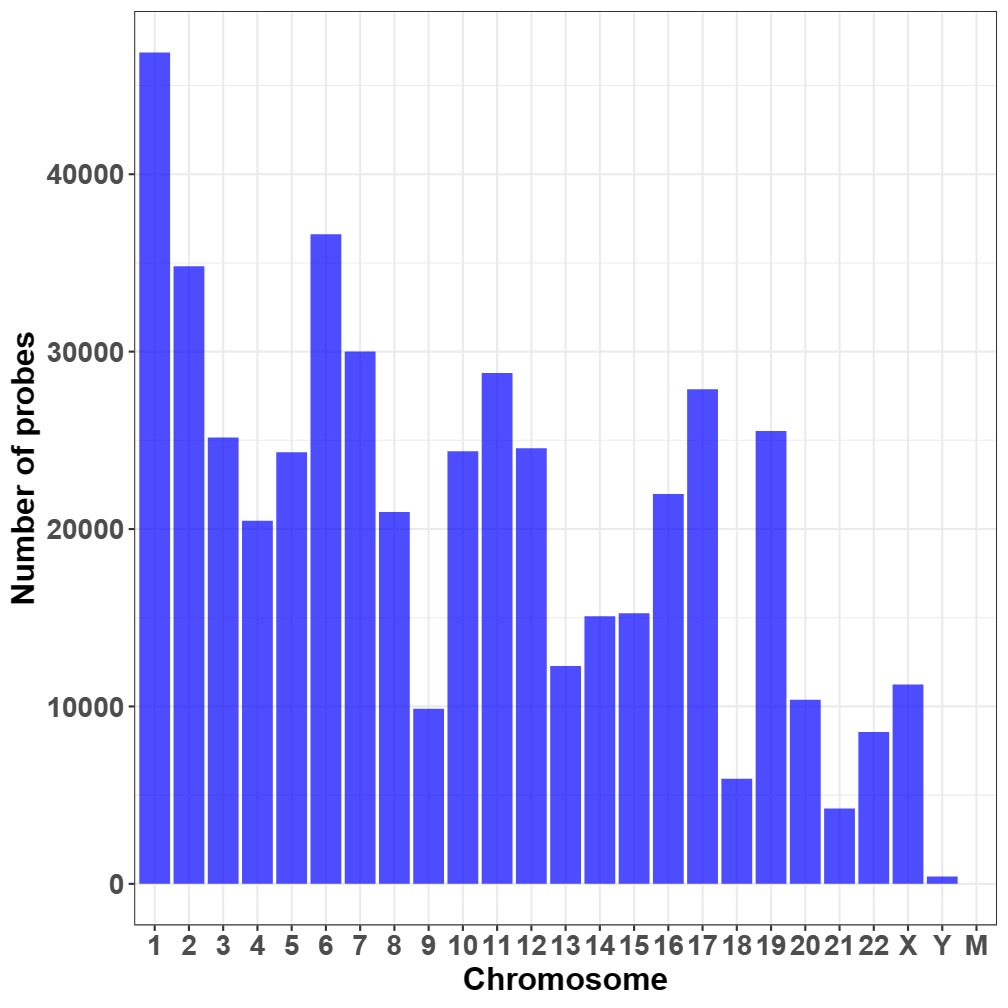


The distribution of 450K methyl probes across chromosomes, data were retrieved from Illumina 450k Bead Arrays integrated in ChAMP tools.

**Supplementary Figure S4**

p < 0.001

p < 0.001

1.
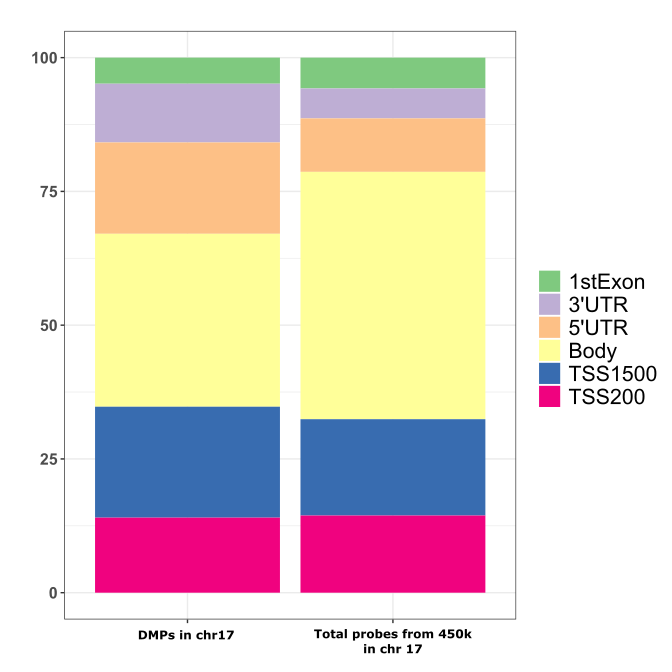
 **B.**
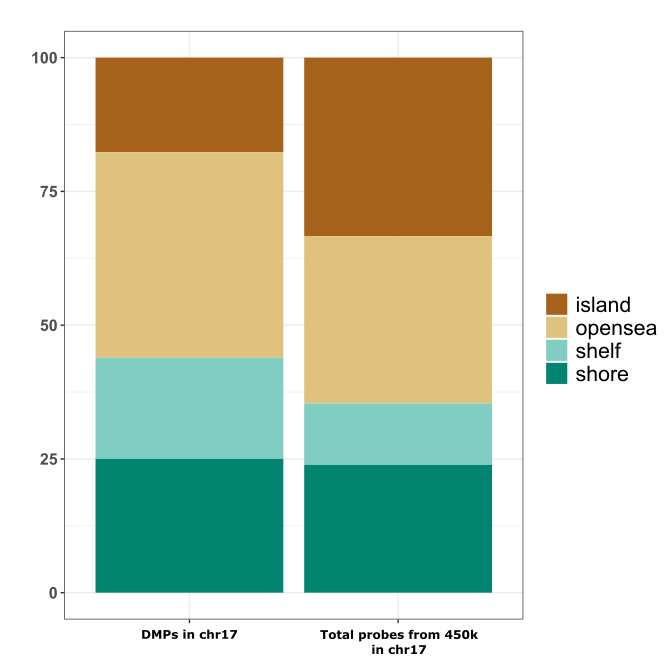


p < 2.2e-16

p < 2.2e-16

**C.
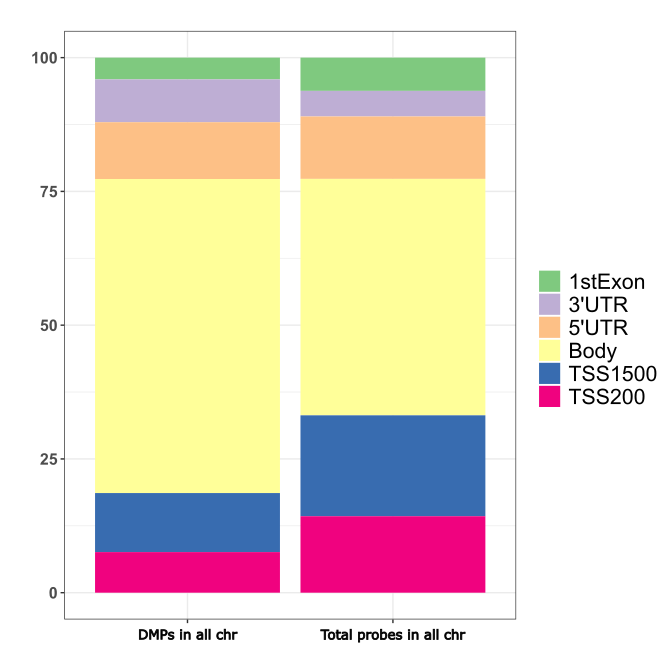
 D.
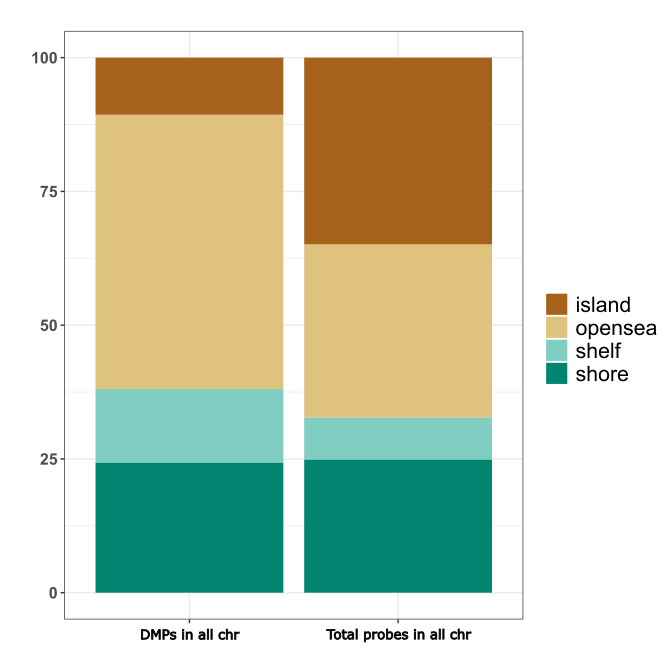
**

Stacked bar charts illustrate the enrichment of DMPs on chromosome 17 (A and B) and the other chromosomes (C and D) in comparison to the designed probes from 450k within the same chromosome, focusing on gene regions and CpG positions.

**Supplementary Figure S5**

**
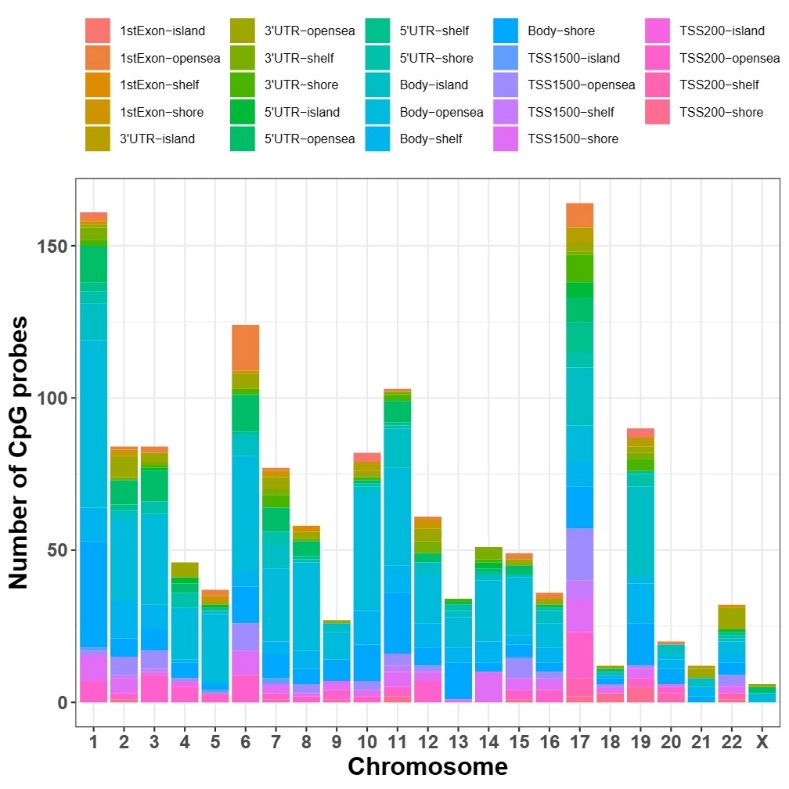
**

The simultaneous distribution of 9060 Differentially Methylated Probes (DMPs) across the combination of gene regions and CpG positions.

**Supplementary Figure S6**


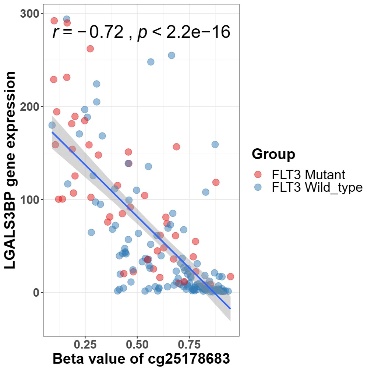

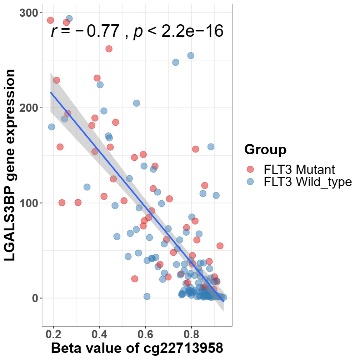

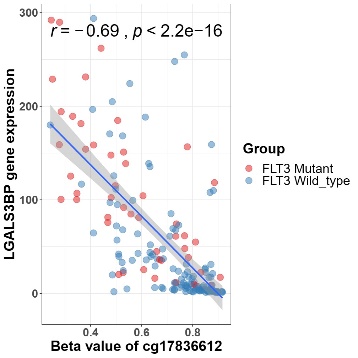

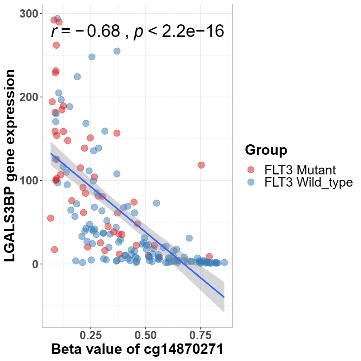

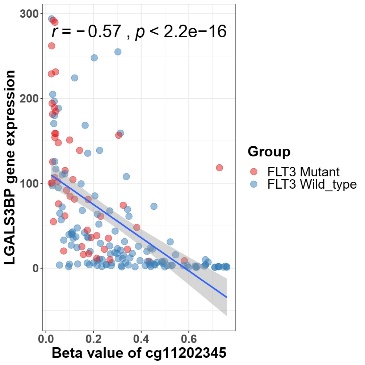

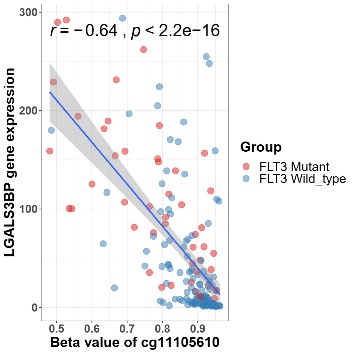

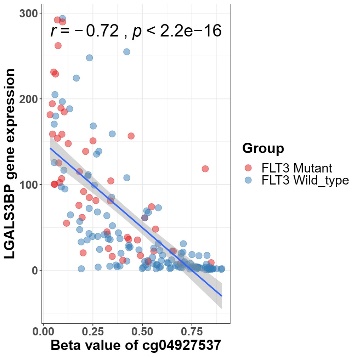


The correlation analysis of LGALS3BP gene expression and the Beta values of DMPs within it.

**Supplementary Figure S7**

**
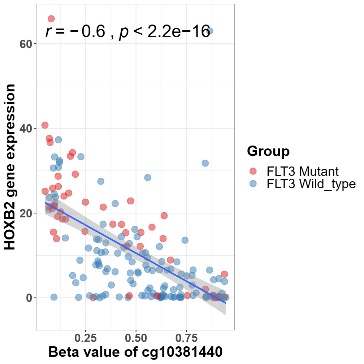

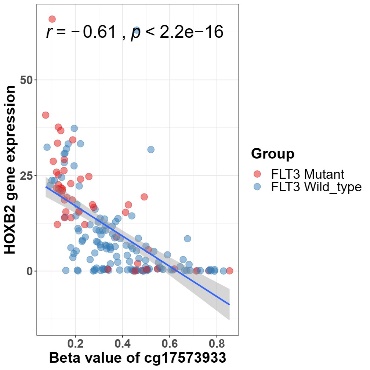

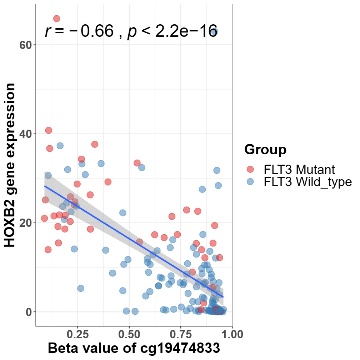

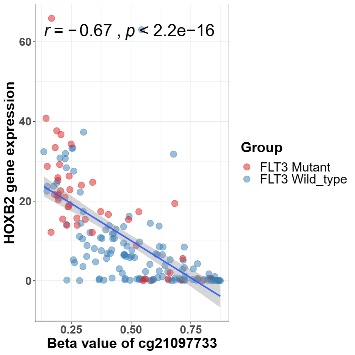
**


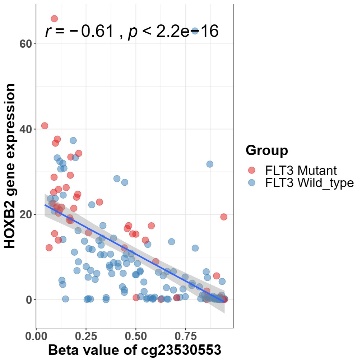

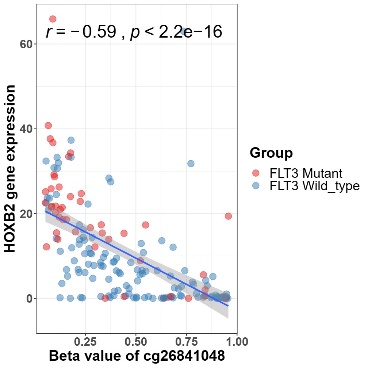


The correlation analysis of HOXB2 gene expression and the Beta values of DMPs within it.

**Supplementary Figure S8**

**
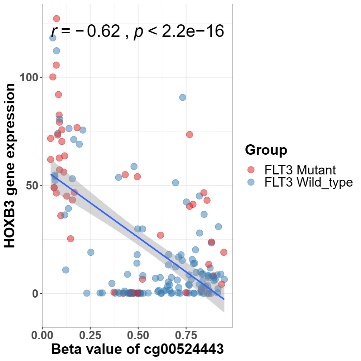

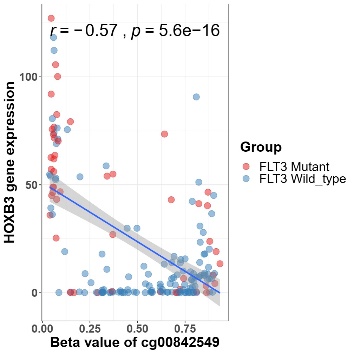

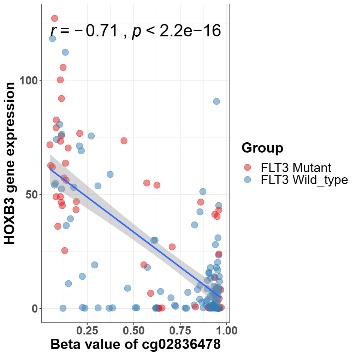

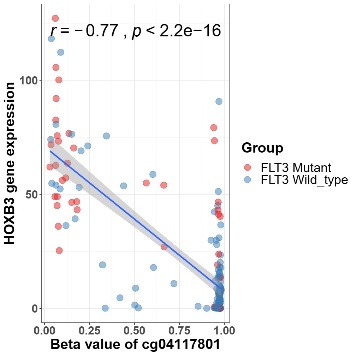
**

**
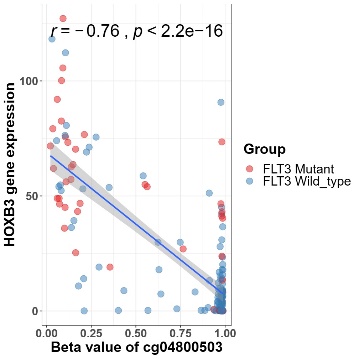

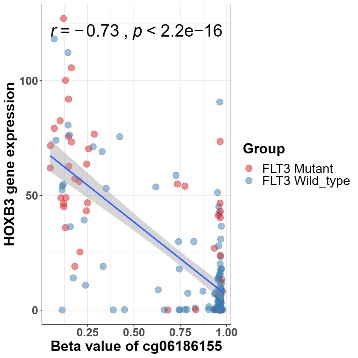

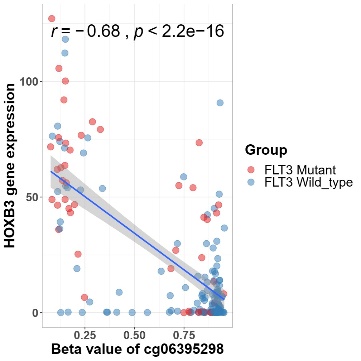

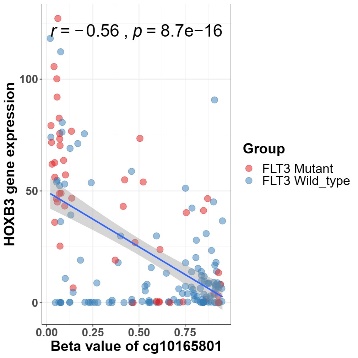

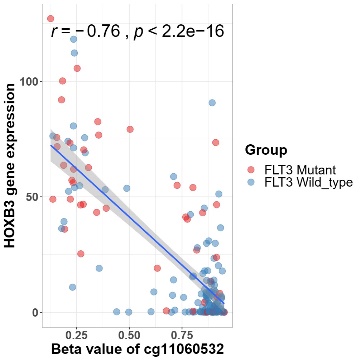

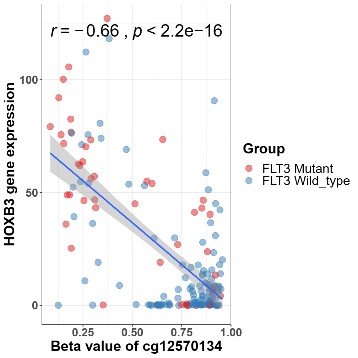

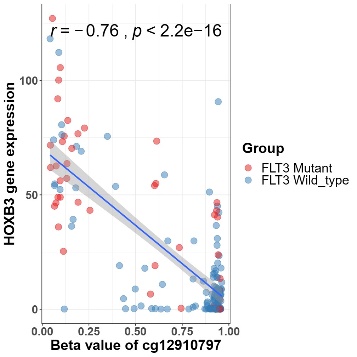

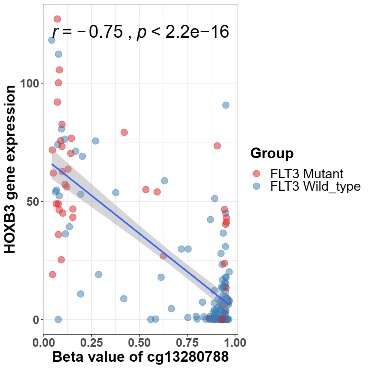
**

The correlation analysis of HOXB3 gene expression and the Beta values of DMPs within it.

**Supplementary Figure S9**

**
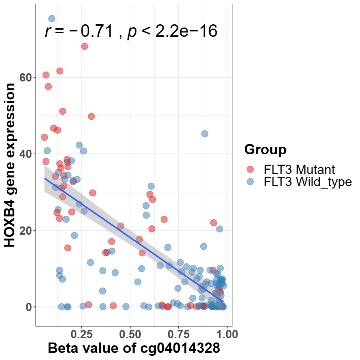

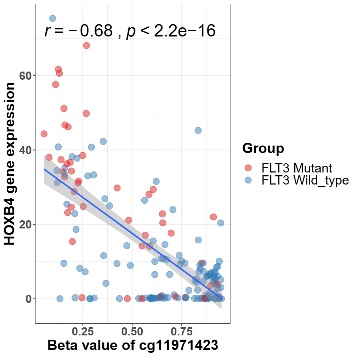

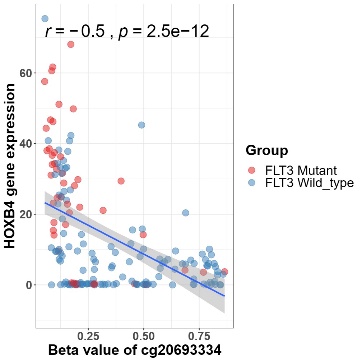

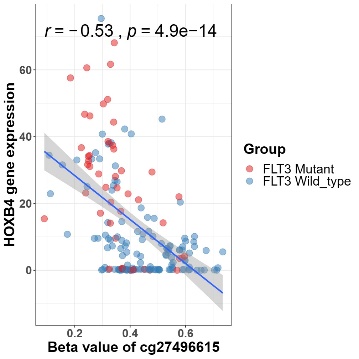
**

The correlation analysis of HOXB4 gene expression and the Beta values of DMPs within it.

**Supplementary Figure S10**

**
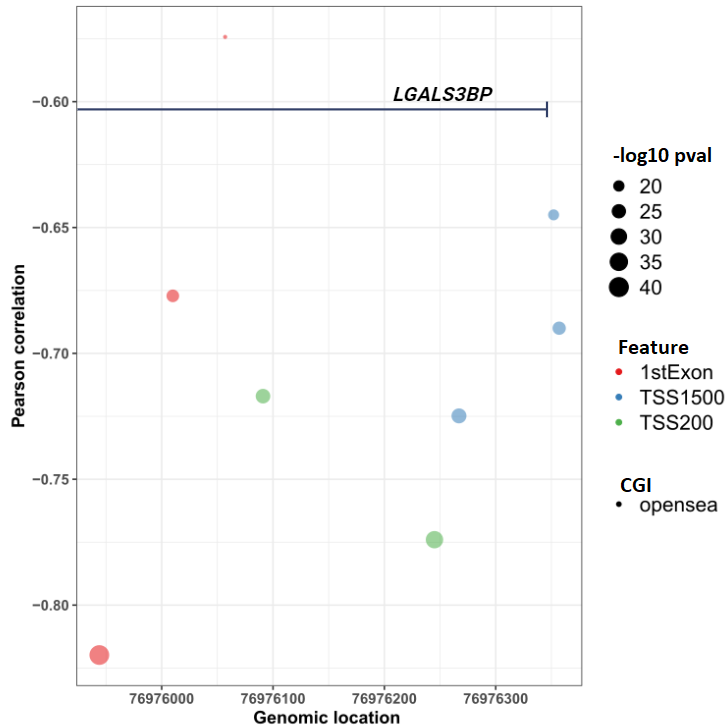

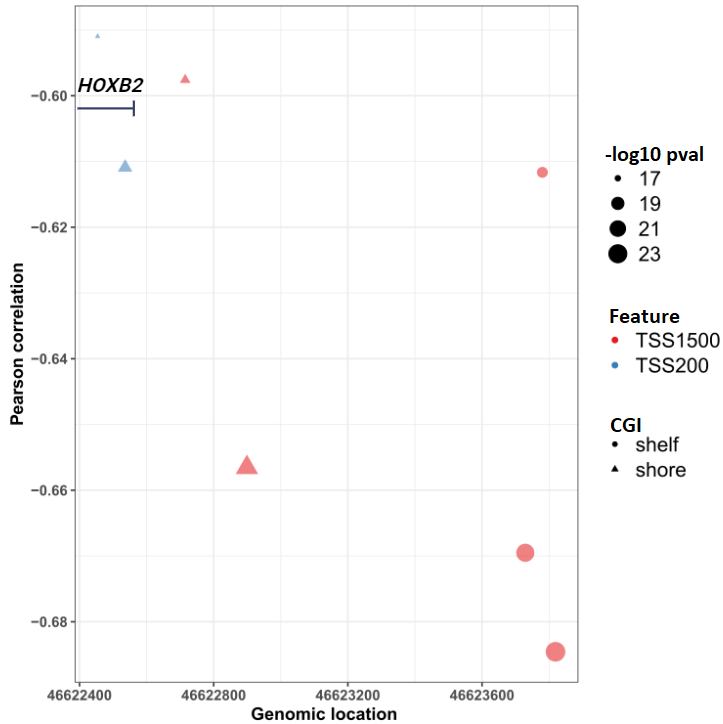
**

**
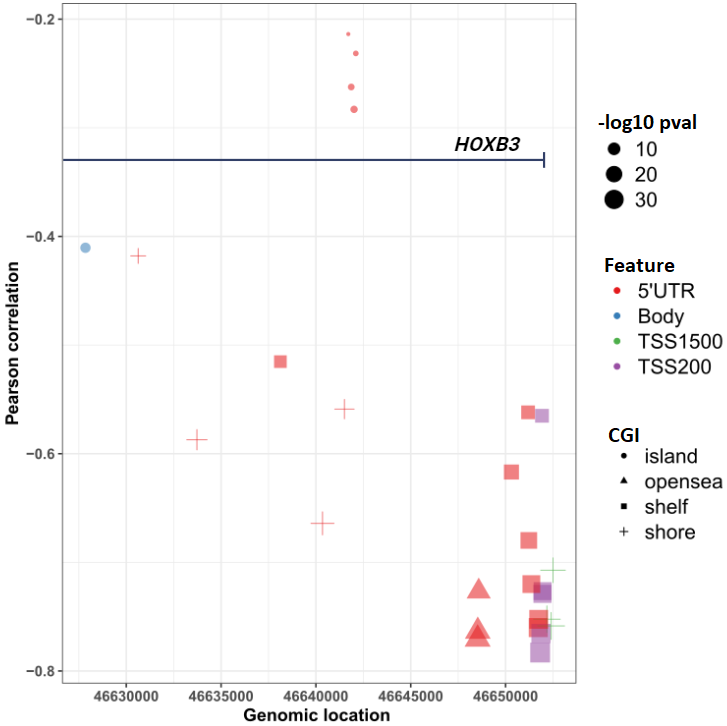

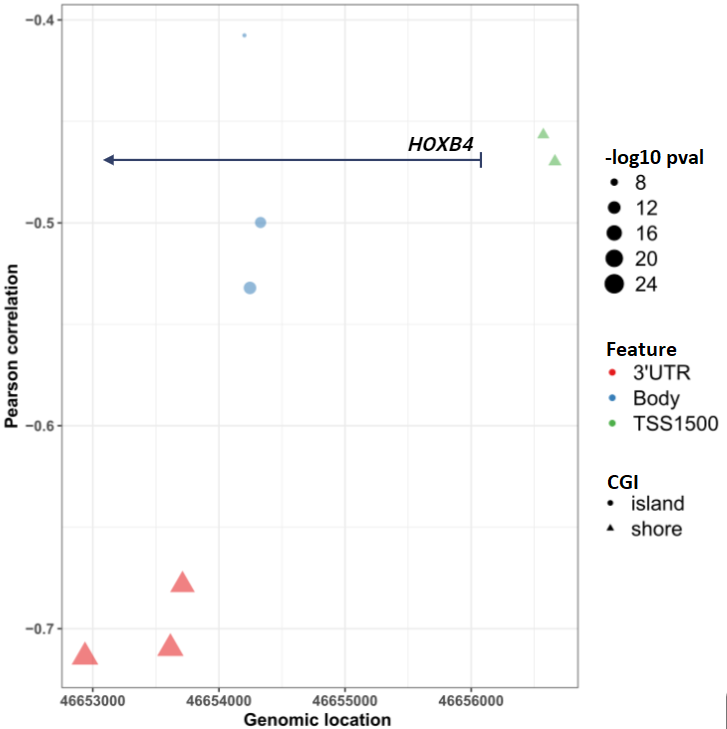
**

Dot plots present the DMPs across their corresponding genes: x-axis = genomic position of the cytosine measured by the probe, y-axis = correlation of methylation to expression (r value), dot size = -log10(p) for correlation, shape = CpG positions, color = gene regions.

**Supplementary Figure S11**

**
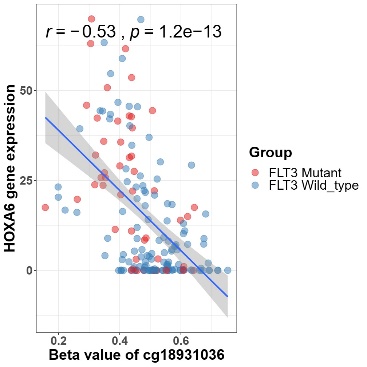

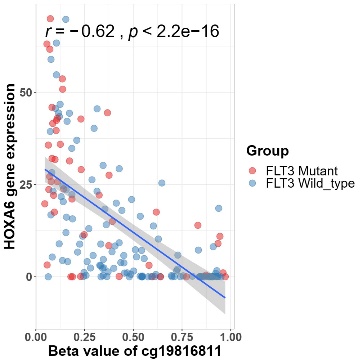

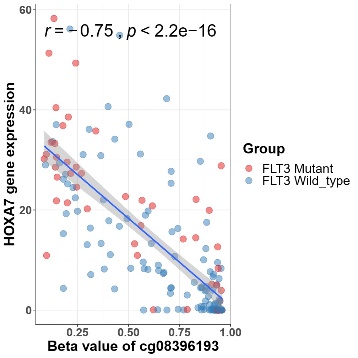

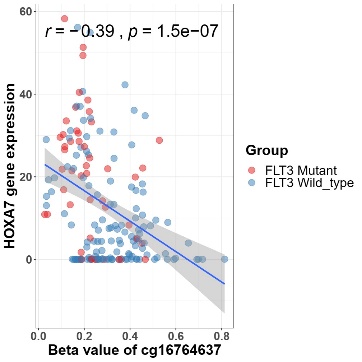

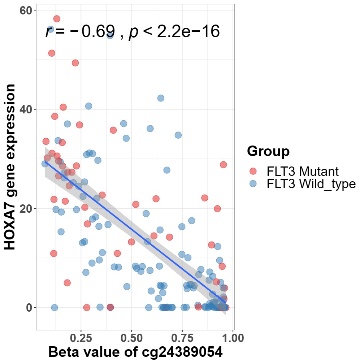

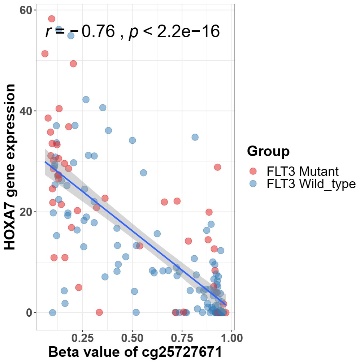

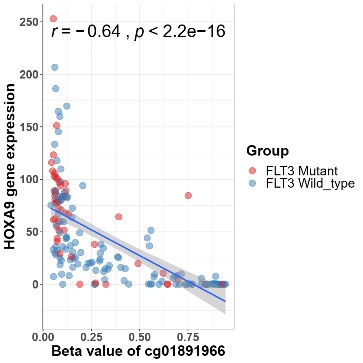

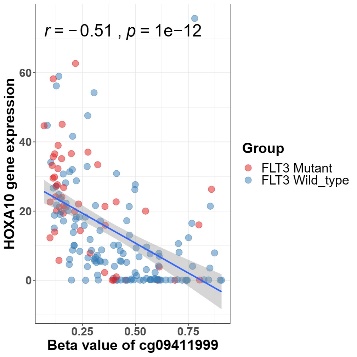
**

The correlation analysis of HOXA gene family gene expression and the Beta values of DMPs within it

**Supplementary Figure S12**

**
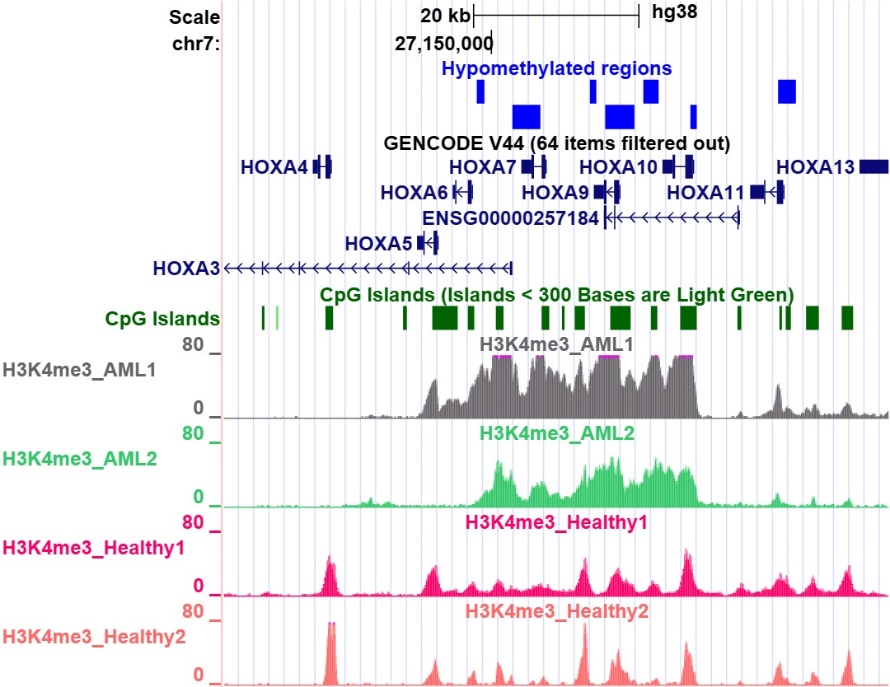
**

Genomic locations of DMRs in relation to H3K4me signals in HOXA gene family

**Supplementary Figure S13**


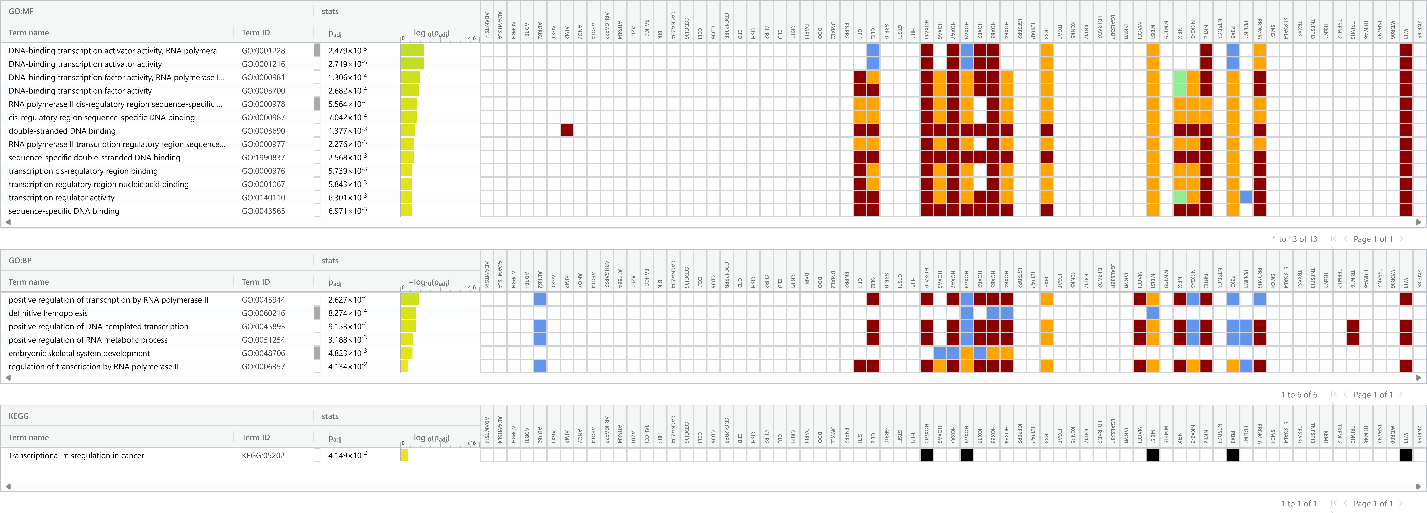


The participation of up-regulated genes in enriched pathways (Adapted from g:Profiler).

Color coded evidence codes for Gene Ontology

: Inferred from experiment or direct assay.

: Expression pattern, Sequence or structural similarity, Genomic context.

: Reviewed computational analysis, Electronic annotation.

: Involved in pathway

HOXA, HOXB gene family and WT1 involved in most of significant pathway related to DNA binding and transcriptional regulation with high evidence. These genes also appeared in KEGG pathway: Transcriptional misregulation in cancer.

**Supplementary Figure S14**

**
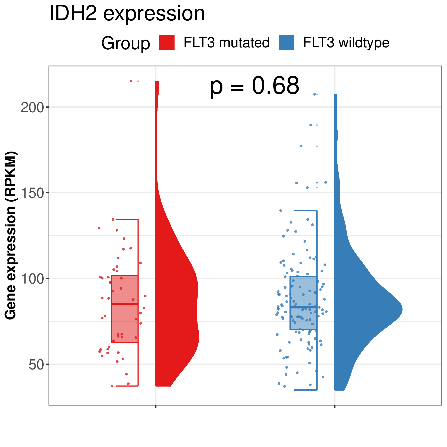

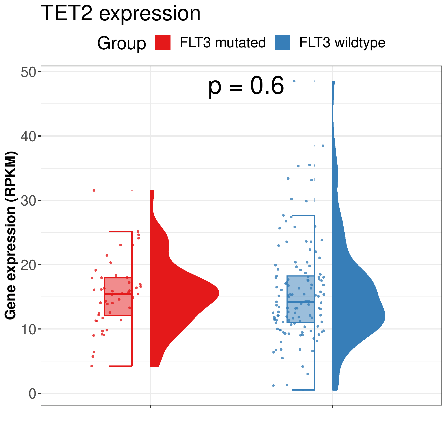
**

Comparative analysis of *IDH2 and TET2* expression between mutant and wildtype groups.

**Supplementary Figure S15**


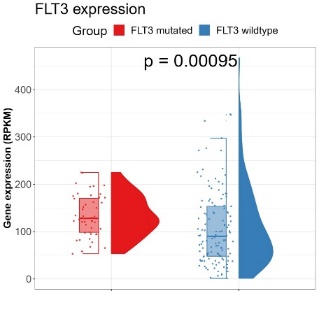

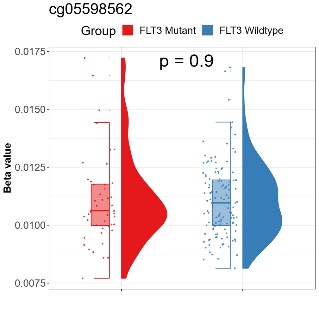

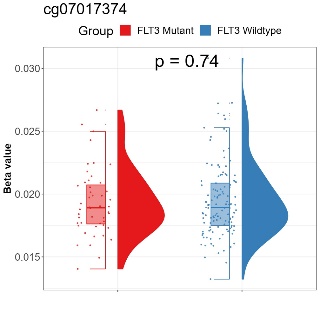

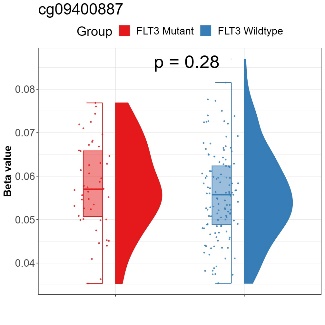

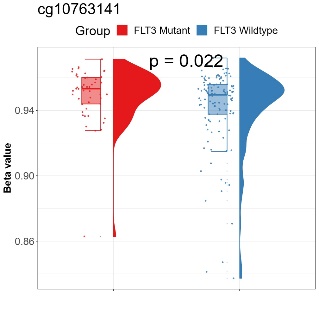

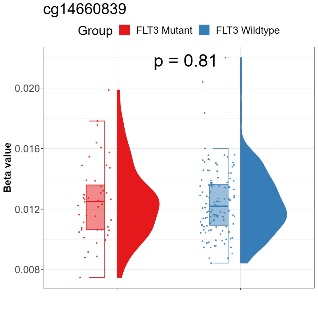

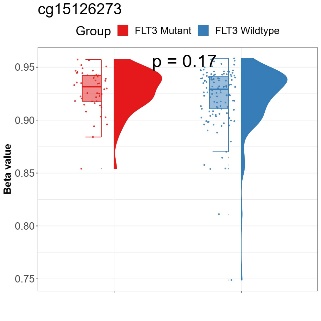

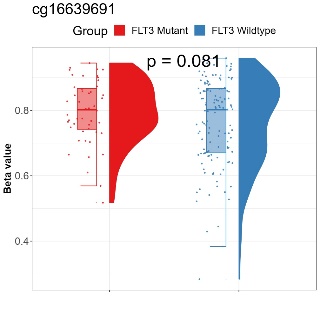


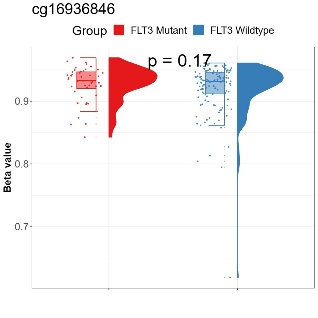

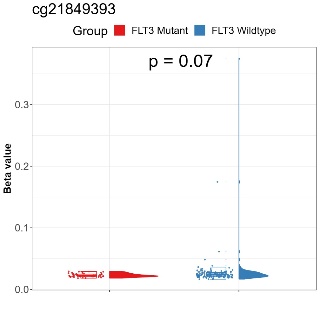

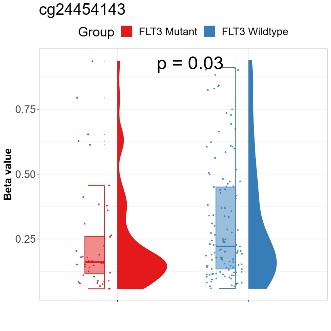

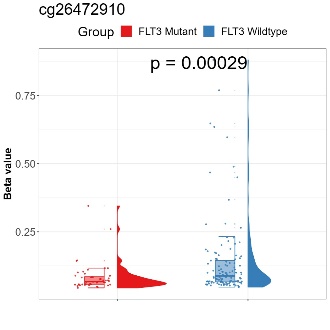


Comparative analysis of *FLT3* expression and its CpG sites beta value between mutant and wildtype groups.


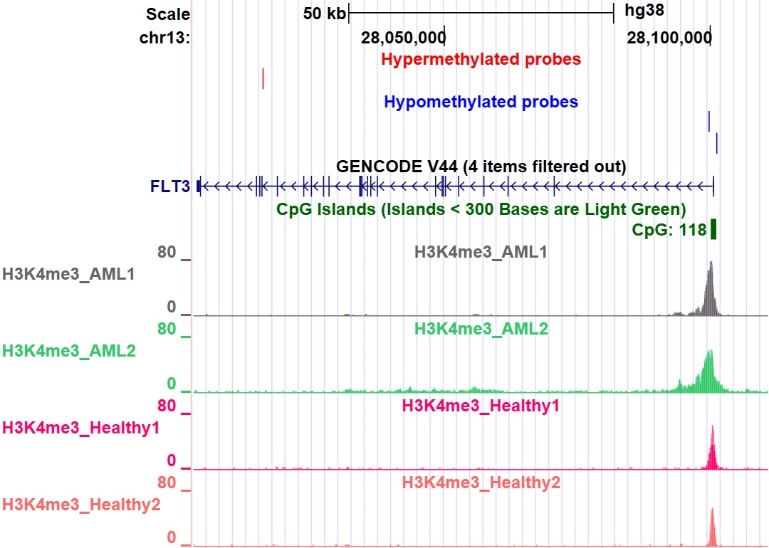


Genomic locations of DMPs in relation to H3K4me signals in *FLT3*

**Supplementary Figure S16**

**
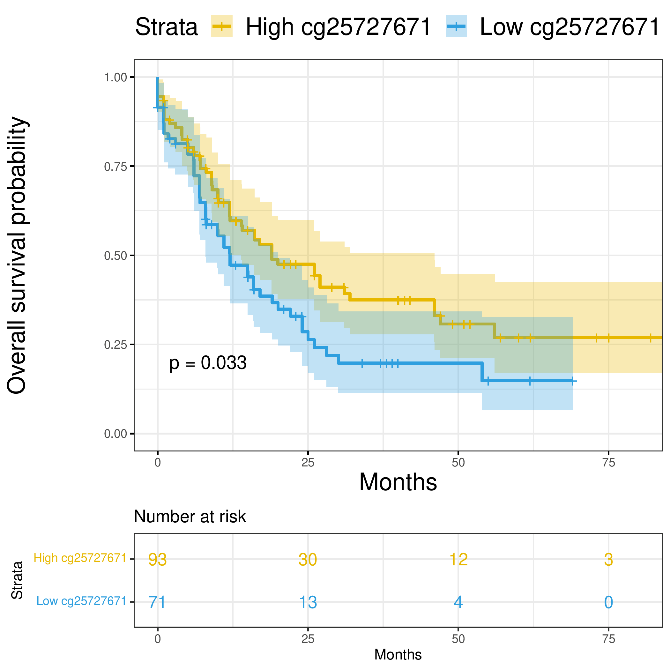

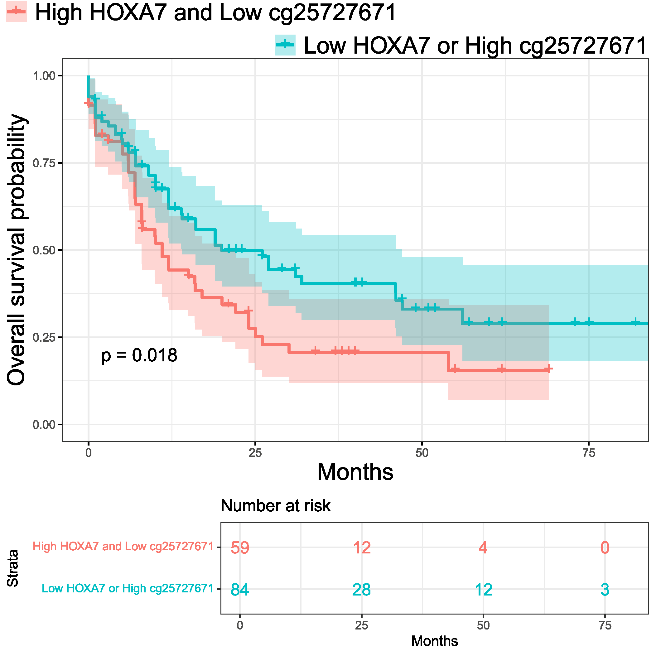
**

Kaplan-Meier survival analysis of *HOXA7’s* CpG site (cg25727671) (left) and survival analysis of combination between this CpG site methylation and *HOXA7* gene expression (right).

**Supplementary Figure S17**

**
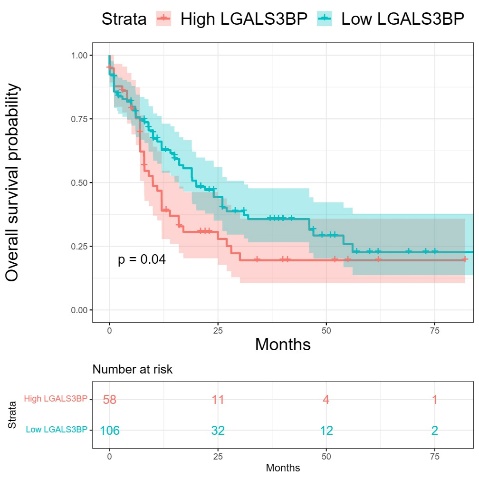

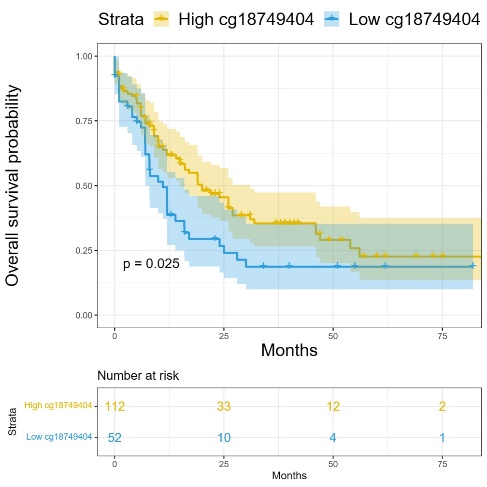
**

Kaplan-Meier survival analysis of *LGALS3BP* categorized by its gene expression (left) and the methylation status of CpG sites within it (right).

**Supplementary Figure S18**

**
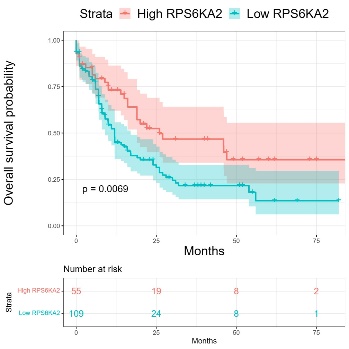

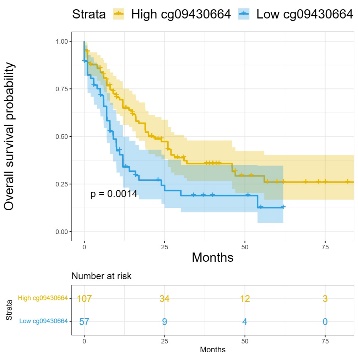

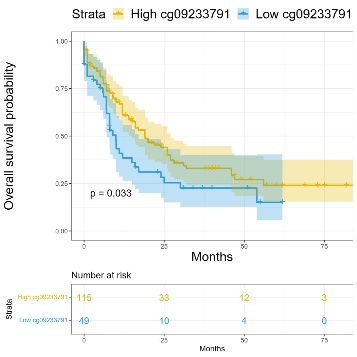

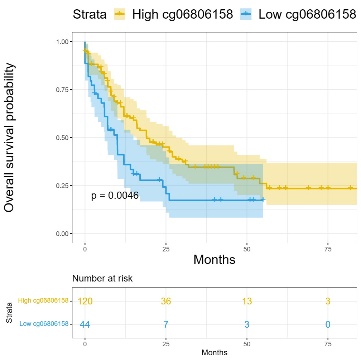
**

Kaplan-Meier survival analysis of *RPS6KA2* categorized by its gene expression (left) and the methylation status of CpG sites within it (right).

**
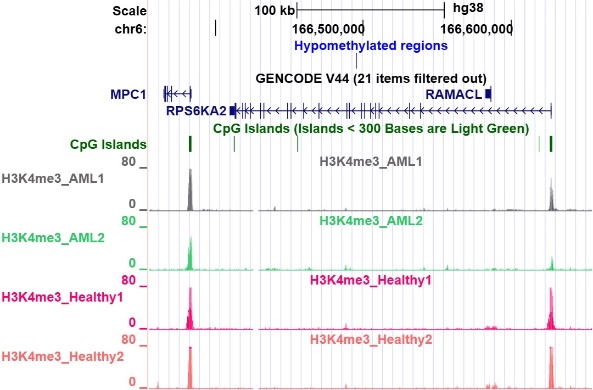
**

Genomic locations of DMRs in relation to H3K4me signals in *RPS6KA2*

**Supplementary Figure S19**

**
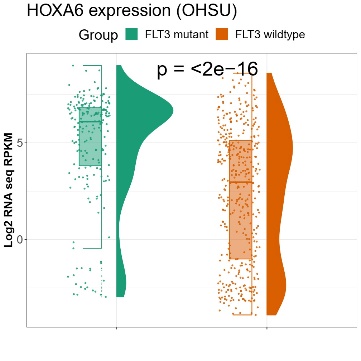

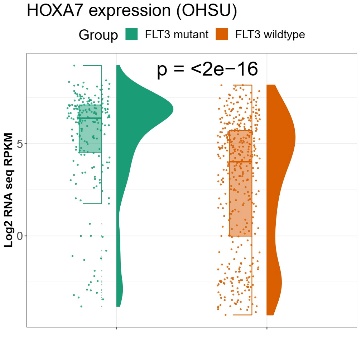

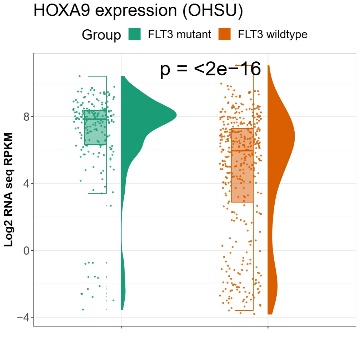

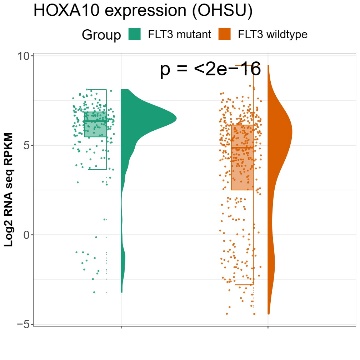
**

**
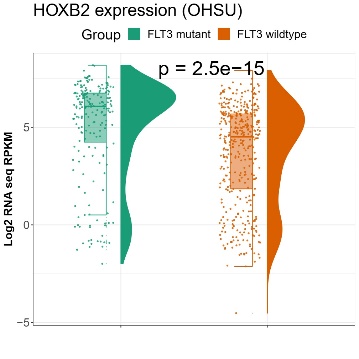

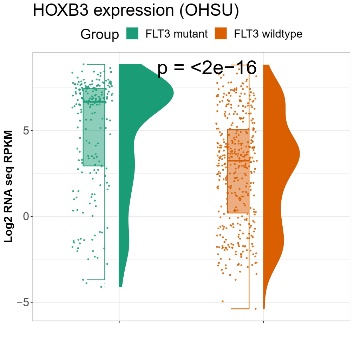

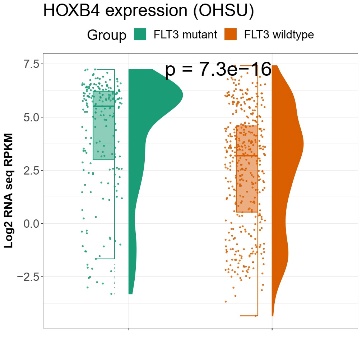

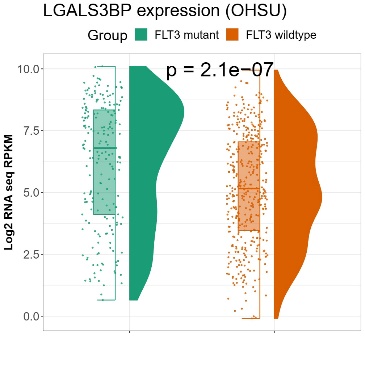
**

**
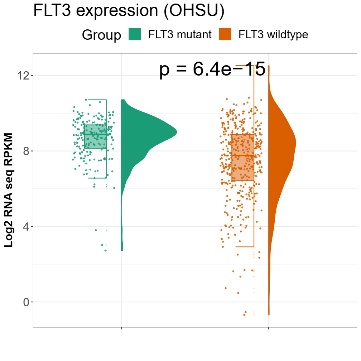

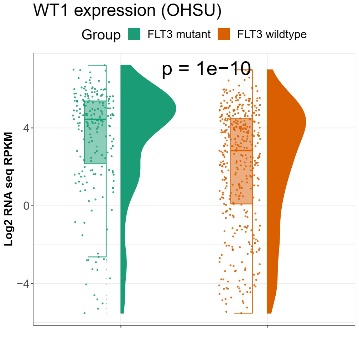

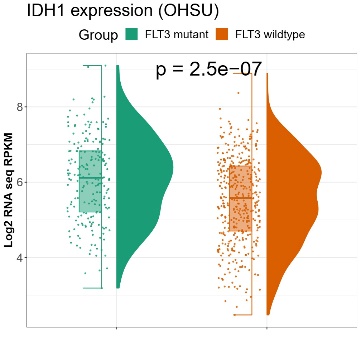

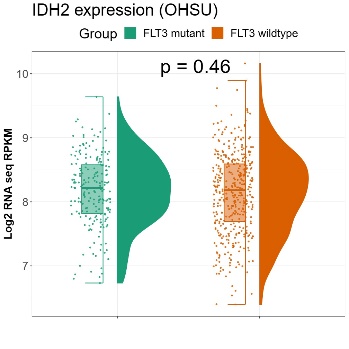
**

**
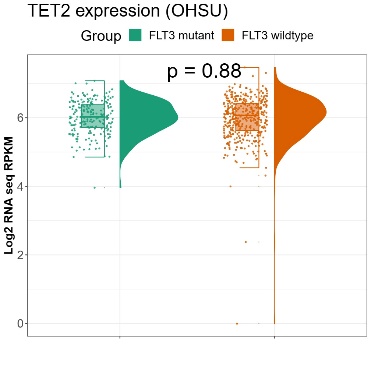
**

Validation of the variance in gene expression of significant genes between the FLT3 mutant and wildtype groups through data obtained from the Beat AML cohort.
